# Exosomal immune decoy integrates cfDNA scavenging and mTOR inhibition for synergistic lupus nephritis therapy

**DOI:** 10.7150/thno.130906

**Published:** 2026-05-11

**Authors:** Mingxin Zhu, Qinyao Xu, Zhicheng Tang, Zexin Wang, Haofang Zhu, Lingyun Sun

**Affiliations:** 1 Department of Rheumatology and Immunology, The Second Affiliated Hospital of Anhui Medical University, Hefei, China.; 2 Department of Rheumatology and Immunology, The First Affiliated Hospital of Anhui Medical University, Hefei, China.; 3 Department of Rheumatology and Immunology, Nanjing Drum Tower Hospital, Affiliated Hospital of Medical School, Nanjing University, Nanjing, China.

**Keywords:** engineered exosomes, cell-free DNA, polylysine, targeted delivery, lupus nephritis

## Abstract

**Objective:**

Lupus nephritis (LN) treatment faces the challenge of balancing effective immunosuppression with systemic safety. To address this, we aimed to develop a biomimetic nanoplatform capable of simultaneously targeting multiple pathogenic pathways in LN, thereby achieving potent immunomodulation without broad toxicity.

**Methods:**

A “smart immune decoy” (RAPA@MEX-PL) was engineered by encapsulating rapamycin (RAPA) in mesenchymal stromal cell-derived exosomes (MEX) and coating the surface with a cationic polylysine (PLL) corona. The platform was designed to concurrently: (1) the polylysine corona potently scavenges cell-free DNA (cfDNA) to quench TLR9-mediated inflammation, (2) the MEX core mediates the repolarization of macrophages from an M1 to an M2 phenotype, and (3) localized RAPA release provides durable mTOR inhibition, synergistically rebalancing autoimmune responses. Renal targeting, immunomodulatory activity, and systemic safety were evaluated in lupus-prone mouse models.

**Results:**

In lupus-prone mice, RAPA@MEX-PL demonstrated precise accumulation in renal tissue, leading to a significant reduction in auto-antibody levels and resolution of glomerular inflammation. The platform concurrently addressed three key pathogenic pathways—cfDNA scavenging, macrophage repolarization and mTOR inhibition—resulting in synergistic rebalancing of autoimmune responses. Notably, it circumvented the metabolic side effects typically associated with systemic RAPA administration.

**Conclusions:**

The RAPA@MEX-PL nanoplatform represents a targeted and effective immunotherapeutic strategy for LN, capable of achieving sufficient immunosuppression without systemic toxicity. These findings emphasize its potential as a favorable candidate for the therapy for autoimmune diseases.

## Introduction

Systemic lupus erythematosus (SLE) manifests as relentless immune dysregulation, with lupus nephritis (LN) affecting 60% of patients and accounting for 10-30% of end-stage renal disease cases 1,2. Current therapies—glucocorticoids, immunosuppressants, and biologics—fail to address two core challenges: halting autoimmune attacks while promoting renal repair 3-5. Despite rapamycin's (RAPA) dual capacity to inhibit mTOR-driven immune activation and mitigate fibrosis, its systemic delivery suffers from off-target effects and poor renal accumulation 6-11. Meanwhile, cell-free DNA (cfDNA) exacerbates inflammation via TLR9/Myd88 signaling, yet no clinical strategy concurrently targets cfDNA clearance and immune modulation 12-16. This therapeutic dilemma underscores the unmet need for a precision medicine approach that integrates immunosuppression, organ protection, and inflammatory resolution.

Mesenchymal stem cell-derived exosomes (MEX) hold great promise as a novel therapeutic modality, capable of recapitulating the immunomodulatory effects of parental cells while circumventing risks associated with live cell transplantation 17-22. Their inherent cargo of regulatory cytokines can attenuate pathogenic immune responses in LN 23-27. However, native MEX lack two critical capabilities: precise targeting to inflamed kidneys and efficient neutralization of circulating cfDNA, a key driver of TLR9-mediated renal inflammation in SLE 28-30. In contrast, cationic polylysine peptides (PLL) offer a powerful electrostatic scaffold that not only binds and neutralizes negatively charged cfDNA with high affinity, but also enhances renal accumulation through charge interactions with the anionic glomerular basement membrane 31-34. While previous approaches have treated cfDNA clearance and immune modulation as separate therapeutic goals 35-39, the synergistic combination between rapamycin inhibition of mTOR, MEX’s immunomodulatory properties, and PLL’s cfDNA-scavenging capacity remains unexplored and uniquely promising for slowing LN progression.

**Figure 1:**
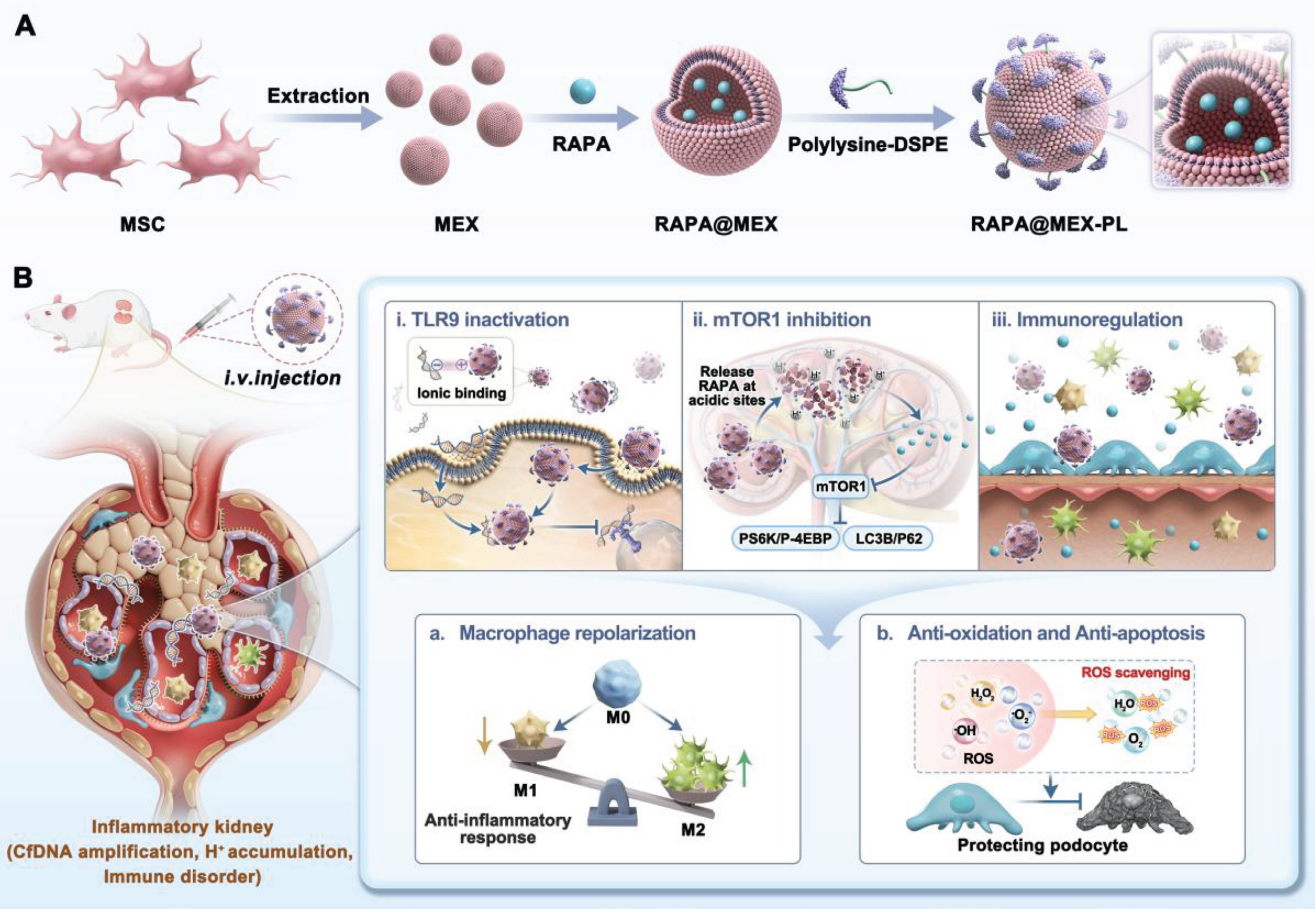
** Schematic illustration of the engineered exosome platform for multi-targeted therapy of LN.** A. Preparation and structural composition of RAPA@MEX-PL, featuring a RAPA-loaded exosome core and a cationic polylysine surface shield. B. The proposed mechanisms of action: (i) Efficient neutralization of circulating cfDNA via electrostatic interaction to disrupt TLR9-mediated inflammation; (ii) Enhanced renal accumulation and localized RAPA release for immunosuppression and tissue protection; (iii) MSC derived exosome-mediated immune regulation reshapes the inflammatory microenvironment. The key functions of exertion in cellular processes: (a) Reprogramming macrophage polarization; (b) Shielding glomerular podocytes from oxidative stress and apoptosis.

Here, we propose a “smart immune decoy” platform by engineering RAPA-loaded MEX with a PLL shield (RAPA@MEX-PL, RMP) for LN treatment (Figure 1). Given that positive charge is critical for mediating the electrostatic interaction between MEX and the glomerular basement membrane, we stably anchored PLL onto the exosomal membrane via DSPE-PEG insertion, forming a positively charged protective corona while preserving exosome integrity. Meanwhile, RAPA was efficiently encapsulated into the exosome using an optimized ultrasound-assisted loading method. This innovative design creates a unique therapeutic synergy that integrates three fundamental mechanisms working in concert: the exosomes’ natural immunomodulatory properties rebalance autoimmune responses while the PLL shield performs dual functions of cfDNA clearance and renal targeting enhancement, complemented by controlled RAPA release for sustained local immunosuppression. These elements form an interconnected therapeutic network where cfDNA neutralization disrupts the inflammation initiation cycle, exosome-mediated immune regulation reshapes the inflammatory microenvironment, and localized RAPA delivery provides durable immunosuppressive effects, collectively establishing a multi-layered defense against LN progression. By simultaneously targeting the disease at multiple levels, this platform represents a transformative approach that moves beyond conventional single-target therapies in LN.

## Materials and Methods

### Material

DSPE-PEG-PLL, Cy5-rapamycin, and DSPE-PEG-FITC were ordered from Ruixi Biotechnology Co., Ltd (Xian, China). DMEM/F12 and fetal bovine serum (FBS) were ordered from Gibco. Phosphate-buffered saline (PBS, pH 7.4) and DMEM were commercially available from the Servicebio Biology Co., Ltd (Wuhan, China). Iscove's Modified Dulbecco's was ordered from Thermo Fisher Scientific (USA). CpG 2006 and its Cy5.5-labeled counterpart were sourced from Genscript China. A 100 μg/mL stock solution of unlabeled CpG was prepared by diluting the compound in PBS. The Annexin V-APC apoptosis kit was acquired from Elabscience. The Bradford Protein Content Assay Kit was sourced from Beyotime Biotechnology Co., Ltd. Additionally, the CCK-8 assay kit, Calcein AM, propidium iodide (PI), Actin-Tracker Green (a microfilament-specific dye), Lysotracker Red DNA-99, 2’, 7’-Dichlorodihydrofluorescein diacetate (DCHF-DA) (for ROS detection), and anti-fade mounting medium with DAPI were all procured from Beyotime Biotechnology Co., Ltd. Rapamycin (RAPA, 10 mM*1 mL in DMSO), calf thymus DNA, Polyethylene glycol 35000 (PEG), puromycin aminonucleoside (PAN), Pam3Cys-Ser-(Lys) 4 (Pam3CSK4), lipopolysaccharide (LPS) and Interferon-gamma (IFN-γ) were obtained from MedChemExpress. Antibodies used for western blot, including CD9, CD81, TSG101, Alix, Histone H10, and Calnexin, were ordered from Abclonal Biological Technology Co., Ltd (Wuhan, China). Antibodies used for flow cytometric analysis were as follows: for *in vitro* experiments, FITC-CD86 and APC-CD206; for *in vivo* experiments, a panel including F4/80-PE, CD86-PE/Cy7, CD11B-FITC, CD206-APC and LY-6G-PerCP/Cy5.5. All antibodies were obtained from BioLegend (USA). Antibodies used for laser confocal microscopy analysis, including anti-CD68, anti-CD206, and anti-iNOS, were obtained from Invitrogen. Antibodies used for immunofluorescence analysis, including pS6K p62 and COL4A3 were obtained from Proteintech, p4EBP and LC3B were obtained from Zenbio. Anti-dsDNA antibody levels (FineTest), enzyme-linked immunosorbent assays (MultiSciences).

### Cells and culture of primary cells

MSCs were supplied by Taisheng Corp (Nanjing) and cultivated in DMEM/F-12 complete medium. Mouse podocyte clone-5 (MPC-5) was obtained from the Servicebio (Wuhan) and incubated in RPMI 1640 media. RAW264.7 was obtained from the Servicebio (Wuhan) and incubated in DMEM high glucose media. Invitrogen (USA) was the source of Ramos Blue™ reporter cells cultured in IMDM complete medium (serum inactivated). The authenticity and functionality of these cells have been confirmed through consistent use in multiple laboratories.

### Isolation and identification of MEX

MSCs at passages 3-8 were first cultured in serum-containing medium. When the cell density reached 80%, the cells were washed twice with PBS following medium removal, and then the culture was switched to serum-free medium. After 3 days of culture, the cell supernatant was harvested and cleared by sequential centrifugation at 4 °C with progressively increasing forces: 300 g (15 min), 2,000 g (20 min), and 10,000 g (30 min). After each centrifugation step, the supernatant was retained, and the pellet was discarded. Concentration of the resulting supernatant was carried out using a 100-kDa molecular weight cut-off (MWCO) ultrafiltration tube (Merck Millipore). To isolate exosomes, the concentrated sample was subjected to centrifugation (12,000 g, 2 h, 4 °C). The recovered exosomes were then resuspended in PBS. The protein content of the exosome preparation was quantified using a protein assay kit, while Dynamic light scattering (DLS) was used to determine particle size and particle number (Zimeng Technology Co., Ltd., China) (About 15 μg exosomes were extracted from 100 ml of serum-free MSC cell supernatant, and the concentration of exosome Particles was 2.5E+9 Particles/mL. The ratio of protein to particle number was 6 μg/10^9^). The morphological structure of exosomes was observed by transmission electron microscope (TEM, FEI Tecnai G2 Spirit, USA). The membrane structure of exosomes was observed by cryo-transmission electron microscopy (cyto-TEM, FEI Talos F200C). The expression of CD9, CD81, Alix, Histone H10, and Calnexin on MEX was analyzed by Western blot.

### Synthesis and characterization of RAPA@MEX-PL

Rapamycin was mixed with MEX (Rapamycin : MEX = 1 : 1, mass ratio) for incubating 10 s. The mixture was then subjected to ultrasonication using a probe-type cell crusher (Xiaomei Ultrasonic Instrument Co., Ltd.). The ultrasonication was performed at 25% of the maximum power output and a frequency of 25 kHz, operating in pulsed mode with 6 cycles of 30 s on and 30 s off. The whole process was conducted in an ice bath to avoid heat-induced damage, subsequently maintained at 37 °C over a 1-hour period to allow membrane recovery. Ultrafiltration centrifugation was used to remove the free RAPA. DSPE-PEG_2k_-PLL was dissolved in 10 mM HEPES at 55 ℃ for 15 s. The resulting suspension was mixed with RAPA@MEX solution for 1 h at 37 ℃ (DSPE-PEG_2k_-PLL : MEX = 2 : 1, mass ratio) to obtain RAPA@MEX-PL. The product was purified using a 1000 kDa ultrafiltration tube. Subsequent characterization included assessment of morphology, particle size distribution, and zeta potential. These analyses were performed by TEM and DLS.

### Quantification of encapsulation efficiency and drug loading capacity

Rapamycin absorbance at 278 nm was recorded on a microplate reader for quantification of drug loading and encapsulation performance. A calibration curve covering 0-50 μg/mL rapamycin was established. Encapsulation efficiency (EE%) was defined as the ratio of encapsulated rapamycin mass to the initial rapamycin mass, expressed as a percentage. Drug loading capacity (DL%) was similarly calculated as the percentage of rapamycin mass relative to the total nanoparticle mass.

### Colocalization of RAPA@MEX-PL

Fluorescent labeling of RAPA, MEX and polylysine (mass ratio, 1 : 1 : 2) was performed using Cy5, DIR and FITC, respectively. Confocal microscopy (Leica, STELLARIS STED) was used to visualize the fluorescently labeled RAPA@MEX-PL.

### RAPA@MEX-PL storage stability

To assess their stability over time, RAPA@MEX-PL (1 mg/ml MEX) were suspended in PBS and stored at 37 ℃ for 7 days, with periodic measurements of their average diameter and zeta potential.

### The release of RAPA from RAPA@MEX-PL *in vitro*

To investigate the drug release profile of rapamycin, RAPA@MEX-PL or free RAPA were separately dissolved in 5 mL of PBS (pH 6.0 or 7.4). The resulting solutions were transferred into dialysis membranes rated for 100 kDa (i.e., retaining molecules above this molecular weight). Immersion of the dialysis bags was carried out using 20 mL of PBS, followed by incubation at 37 °C with orbital shaking at 80 rpm. At predetermined time points, 1 mL of the external buffer (outside the dialysis membrane) was withdrawn to measure the drug concentration using a multimode microplate reader (Thermo Fisher). After each sampling, 1 mL of fresh PBS was replenished to maintain a constant volume.

### Evaluation of cfDNA-binding efficiency of engineered exosomes

Separate solutions of calf thymus DNA and ethidium bromide (EtBr) were prepared in PBS at 1 mg/mL each. A reaction mixture was prepared by combining the following components: calf thymus DNA (4 μL), EtBr (4 μL), specified volumes of RMP, RM, MEX, or PEG, and either FBS or PBS (16 μL). The final volume was adjusted to 160 μL with PBS. Incubation of the mixture proceeded at 37 °C over 24 h. The supernatant (100 μL) was then gently transferred to a new 96-well plate, and its fluorescence signal was recorded using a multimode microplate reader (Thermo Fisher; λ_ex_ = 485 nm, λ_em_ = 590 nm). The nucleic acid binding efficiency of the cationic materials was quantified according to the equation below. Binding efficiency (%) = [(1-(A-A_0_/A_1_-A_0_)] × 100%. Fluorescence intensity was measured for three samples: (i) the EtBr/DNA complex present in the supernatant after incubation (termed A); (ii) free EtBr (termed A_0_); and (iii) the untreated EtBr/DNA complex (termed A_1_).

### To test the cfDNA-binding efficacy

Cationic materials were first incubated with CpG (0.4 mg/mL) at 4 °C for 24 h. Bromophenol blue (0.25%) was added to the resulting mixture, which was then separated by electrophoresis on a 2% agarose gel. Electrophoresis was carried out at 120 V for 40-45 min. Gel visualization was achieved with a WB600Pro imaging system. (BLT Biotechnology Co., Ltd.).

### Extracellular agonist-mediated suppression of Toll-like receptor activation

The ability of engineered exosomes to inhibit TLR9 activation was evaluated using Ramos Blue™ reporter cells. Cells were seeded in 96-well plates at a density of 5 × 10⁴ cells per well. After cell attachment, Pam3CSK4 (1 µM) and CpG-ODN2006 (1 µM) were mixed with different components and then added to the 96-well plate. Following incubation at 37 °C for 24 h, the supernatant was collected. The QUANTI-Blue assay was used to assess SEAP (secreted embryonic alkaline phosphatase) activity, following the manufacturer's instructions. The supernatant was combined with the detection medium. Following a 1-hour incubation at room temperature, the absorbance was read at 650 nm using a multimode microplate reader. TLR activation was expressed as (X-X_0_)/(X_1_-X_0_) × 100%, with X, X_0_, and X_1_ corresponding to the optical density of the test, control, and agonist-only groups, respectively.

### Cellular colocalization of CpG and engineered exosomes

One day prior to the experiment, confocal culture dishes were seeded with RAW264.7 cells at a concentration of 2 × 10⁴ cells per dish. On the following day, two parallel experiments were performed. In the first experiment, two fluorescent probes were added simultaneously: Quasar 670-conjugated CpG-2006 (1 µM) and exosomes labeled with FITC. This was done to assess whether the engineered exosomes could reduce cellular uptake of cfDNA. In the second experiment, cells were subjected to an initial 4-hour incubation. During this time, they were exposed to Quasar 670-labeled CpG-2006 at a concentration of 1 µM., followed by the addition of FITC-labeled engineered exosomes to evaluate whether the engineered exosomes could bind to intracellularly internalized CpG. At 4, 8, and 12 h time points, the incubation was terminated. Lysosomes were visualized by staining with LysoTracker Red DND-99. Nuclei were counterstained separately with DAPI. Imaging was performed using a confocal microscope (Leica STELLARIS STED).

### The CCK-8 assay

Briefly, RAW264.7 cells were prepared for the experiment by seeding them into 96-well plates. The seeding density was 1 × 10⁵ cells per well, and this was done on the evening before the experiment. After overnight culture, the original medium was replaced with fresh complete medium containing graded concentrations of rapamycin or engineered exosomes. Cells were then incubated for a further 24 h or 48 h, after which the medium was removed and CCK-8 working solution was added for viability assessment. The absorbance of each well was recorded at 450 nm using a multimode microplate reader (Thermo Fisher).

### The Calcein/PI cell cytotoxicity assay

After seeding and attachment in 24-well plates, the culture medium was replaced with complete medium containing the indicated treatments and incubated for 24 h. Subsequently, preparation of the dye solution followed the manufacturer’s guidelines, after which it was kept in darkness for a 30-minute incubation. Cell images were captured using a Nikon Ti2-A microscope.

### *In vitro* cellular uptake study

Coverslips were placed in 12-well plates, and RAW264.7 and MPC-5 cells were used for seeding. The cell density was adjusted to 1 × 10⁶ cells per well. RAW264.7 cells were stimulated with 10 µg/mL LPS (induces M1 polarization) and 20 ng/mL IFN-γ (synergizes with LPS for M1 polarization) for 24 h. MPC-5 cells were divided into two groups. One group was stimulated with PAN (15 µg/mL), which induces podocyte injury. The other group served as untreated controls. 24 h later, they were incubated for 2 h with Cy5-labeled formulations: RAPA (10 µM), RAPA@MEX (9.55 μg/ml MEX, 10 µM RAPA), and RAPA@MEX-PL (9.55 μg/ml MEX, 10 µM RAPA). Following PBS washes, Cy5 internalization was quantified by flow cytometry (CytoFLEX, Beckman Coulter, USA). In parallel, nuclei were stained with DAPI for confocal imaging (Leica STELLARIS STED).

### Macrophage polarization

RAW264.7 cells were treated as described above to induce M1 polarization. Following a 24-hour exposure to the drug, the culture supernatant was aspirated, and the cell monolayer was gently rinsed twice with PBS. Subsequently, fixation was performed using 4% paraformaldehyde for 15 min at ambient temperature, followed by a 10-minute permeabilization step with 0.3% Triton X-100. After two additional PBS washes, non-specific binding was blocked by incubating the cells with 5% FBS for 30 min. The cells were then incubated overnight at 4 °C with primary antibodies targeting iNOS, CD206, and CD68. On the following day, secondary antibody incubation was carried out for 1 h at room temperature. Nuclear counterstaining was achieved with DAPI. Fluorescence images were acquired using a confocal microscope. For flow cytometric analysis, cells were detached and subjected to a single PBS wash. Cells were then stained with a live/dead viability dye for 20 min, and the reaction was terminated with PBS. After centrifugation, cells were surface-stained with a prepared solution containing CD86 antibody for 20 min, followed by PBS termination and centrifugation. Cells were then fixed and permeabilized, followed by intracellular staining with a prepared solution containing CD206 antibody for 30 min. PBS was used to terminate the reaction, after which the cells were resuspended in 200 μL of the same buffer and immediately analyzed on a CytoFLEX instrument (Beckman Coulter, USA). FlowJo software (FlowJo, USA) was used for data analysis.

### Assessment of mTOR and autophagy signaling pathways

RAW264.7 cells, following polarization induction and a 24 h drug exposure. Fixation of the cells was carried out with 4% paraformaldehyde for 15 min at ambient temperature. Permeabilization was subsequently achieved using 0.3% Triton X-100 over a 10-minute period. Overnight incubation at 4 ℃ was conducted using primary antibodies directed against pS6K and p4EBP1 (mTOR signaling) or p62 and LC3B (autophagic flux). After two washes in PBS, the samples were treated with secondary antibodies (1 h, room temperature). Nuclear counterstaining followed using DAPI. The dye contained an anti-fade reagent. Imaging was performed on a Leica STELLARIS STED confocal microscope. The expression of p62 and LC3B (autophagy flux) on RAW264.7 cells were also analyzed by Western blot.

### The cell apoptosis assay

For the experiment, MPC-5 cells were prepared in 6-well plates. This cell seeding was carried out one day in advance. After cell attachment, the culture medium was supplemented with PAN at a concentration of 15 μg/mL together with engineered exosomes at indicated concentrations. Following a 24 h treatment, the apoptosis rate was assessed. Staining was carried out as per the manufacturer’s protocol. Subsequently, the samples were analyzed on a flow cytometer.

### Measurement of ROS

The culture medium was first removed. Subsequently, the cells were gently rinsed with warm PBS. Preparation of 10 μmol/L DCFH-DA was performed according to the manufacturer's directions. The probe was then incubated with the cells at 37 °C for 30 min, and the cells were subsequently rinsed three times using warm buffer, and then imaged with a fluorescence microscope (Ti2-A, Nikon). The resulting fluorescence intensity was quantified using ImageJ software.

### Detection of F-actin

Following a 24 h co-treatment with 15 µg/mL PAN and engineered exosomes, the cells were subjected to cytoskeleton staining. To visualize the cytoskeleton, MPC-5 cells were fixed at room temperature for 15 min and then stained with Actin-Tracker Green, which specifically labels microfilaments. DAPI staining was then carried out for 10 min. Fluorescence and DAPI signals were subsequently detected using a Leica STELLARIS STED confocal microscope.

### Biodistribution of RAPA@MEX-PL *in vivo*

To evaluate the biodistribution of the different formulations, RAPA, RAPA@MEX, and RAPA@MEX-PL were labeled with Cy5 and excess dye or antibody was removed by ultrafiltration (3 cycles). The dose of RAPA was 1 mg/kg in all formulations. For the nanoparticle groups, the MEX carrier dose was 10.50 mg/kg. The labeled drug was injected intravenously into MRL/lpr and MRL/MPJ mice. An AniView Pro animal imaging system (BLT Biotechnology Co., Ltd.) enabled tracking of drug biodistribution via NIRF (near-infrared fluorescence) at designated time points across a 24-hour window. At 4 h and 24 h post-administration, the animals were euthanized, and their principal organs were collected for ex vivo NIRF imaging. The imaging system’s native software provided a tool for fluorescence quantification. Using this tool, we determined the average intensity per sample. In addition, OCT embedding and frozen sectioning of fresh heart, lung, liver, kidney, lymph nodes, and spleen were performed using an HM525 NX automatic cryostat (Epredia). Meanwhile, renal tissue sections were stained overnight with COL4A3 primary antibody, incubated with secondary antibody, and nucleated with DAPI. Tissue sections were visualized with a Leica STELLARIS STED confocal microscope following 10 min of DAPI staining.

### MRL/lpr spontaneous SLE mouse model

MRL/lpr mice (16 weeks old) were randomized into 8 groups: 7 treatment groups receiving weekly IV injections of PBS, MSC, MEX, RAPA, MEX-PL, RAPA@MEX (RM), or RAPA@MEX-PL (RMP) for 4 weeks, plus one healthy control (MRL/MPJ). The dose of RAPA was 1 mg/kg in all formulations. For the nanoparticle groups, the MEX carrier dose was 10.50 mg/kg. Body weight was monitored weekly. Post-treatment, urinary protein was quantified via BCA assay. Spleen and axillary lymph nodes were weighed. Kidneys underwent H&E and immunohistochemical staining. Blood was analyzed for hepatic/renal function, serum creatinine, anti-dsDNA antibodies, and cytokines (ELISA). Splenocytes were analyzed by flow cytometry using F4/80-PE, CD86-PE/Cy7, CD11B-FITC, CD206-APC, and LY-6G-PerCP/Cy5.5. The Animal Ethics Committee of the First Affiliated Hospital of Anhui Medical University granted approval for all animal procedures (Approval No. IACUC-2502029).

### Extraction and measurement of cfDNA

CfDNA was isolated from mouse serum (MRL/MPJ and MRL/lpr mice) with a commercial nucleic acid extraction kit. The concentration of the isolated cfDNA was subsequently determined using a Nanodrop 2000 spectrophotometer (Thermo Fisher).

### Evaluation of the biocompatibility of RAPA@MEX-PL

After the treatment, blood samples were collected to measure the levels of liver functions. Potential pathological damage to the major organs was assessed by H&E staining.

### Evaluation of renal histopathology

The renal tissue was examined under a light microscope to assess glomerulonephritis through H&E staining. To visualize and quantify immune complex deposition, tissue sections were stained overnight with primary antibodies (mouse IgG, C3, nephrin or WT-1), followed by secondary antibody incubation and DAPI nuclear counterstaining. Quantitative analysis of glomerular fluorescence intensity for IgG (red) and C3 (green), nephrin (red) and WT-1 (green) was performed using ImageJ software.

### Statistical analysis

Biological replicates (n ≥ 3) were incorporated into all experimental conditions. Data are mean ± SD, analyzed with GraphPad Prism 9.0. For comparisons between groups, one-way ANOVA with Tukey's correction was applied. Significance levels: *p < 0.05, **p < 0.01, ***p < 0.001.

## Results and Discussion

### Synthesis and characterization of RAPA@MEX-PL

**Figure 2:**
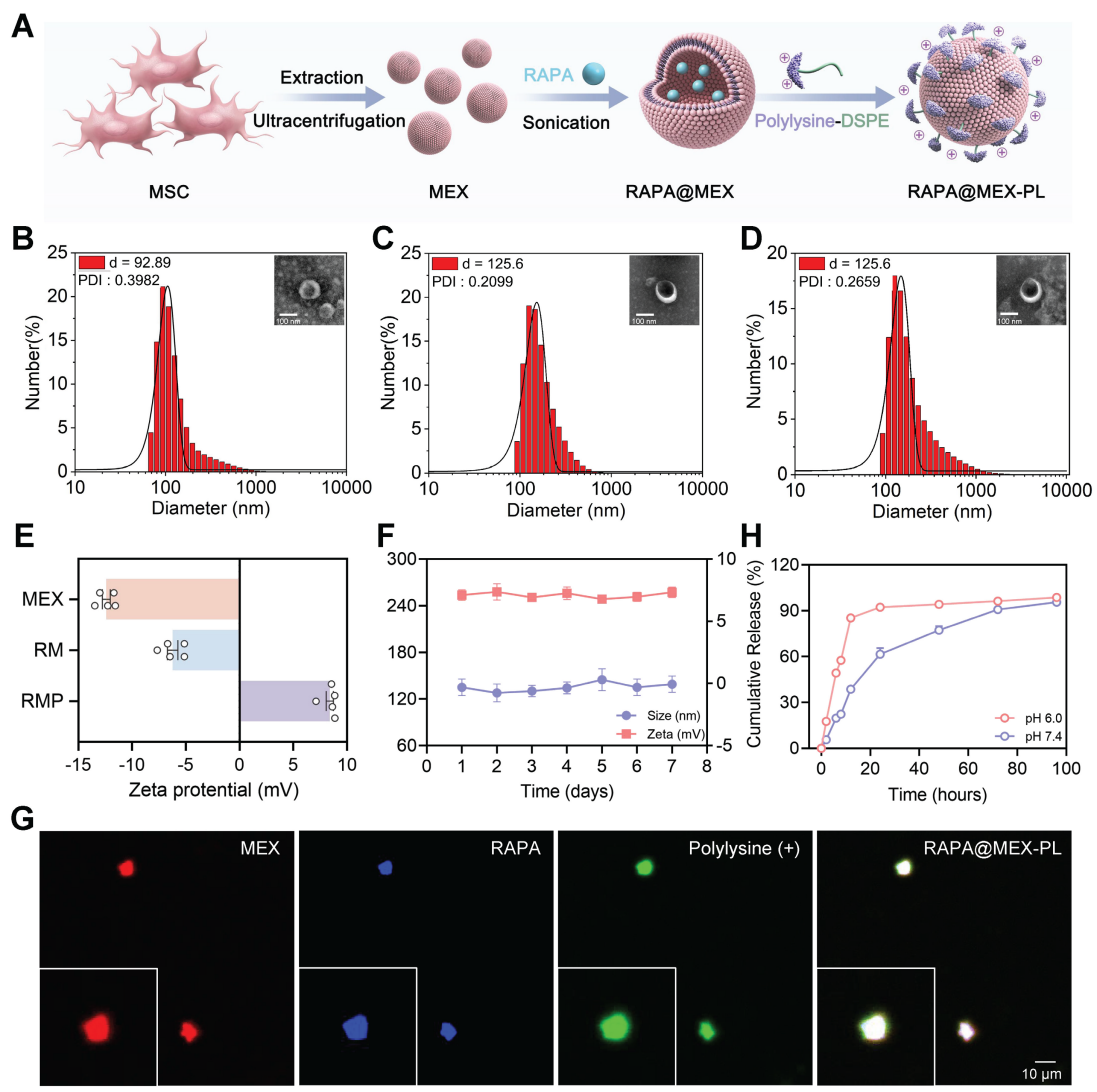
** Fabrication and characterization of RAPA@MEX-PL.** (A) Schematic depicting the fabrication process of engineered exosomes. (B-D) Representative Transmission electron microscope (TEM) images and corresponding hydrodynamic size distribution (insets) of (B) MEX, (C) RM and (D) RMP. Scale bar, 100 nm. (E) Average zeta potential of MEX, RM and RMP (mean ± SD, n = 5). (F) The stability of RMP was assessed by monitoring its size and zeta potential after incubation in PBS at 37 ℃ for 7 days (mean ± SD, n = 5). (G) Confocal fluorescence microscopy images of the assembled RMP, showing the colocalization of MEX membrane (red), RAPA (blue) and DSPE-PEG-PLL (green). Scale bar, 10 μm. (H) Drug release efficiency of RMP in different pH solutions (mean ± SD, n = 5).

To materialize the proposed “smart immune decoy” strategy, we engineered a cationic exosome platform, RMP, through a rational and modular fabrication process (Figure 2A). MEX served as the innate immunomodulatory core, which were first loaded with RAPA via an optimized ultrasound method. This technique achieved a remarkably high encapsulation efficiency of 95.25% and a drug-loading efficiency of 24.10% at a 1 : 1 mass ratio, underscoring its efficacy in leveraging the exosomal lipid bilayer for hydrophobic drug carriage. Subsequently, a positively charged PLL corona was stably anchored onto the exosomal membrane using DSPE-PEG-PLL, a lipid-polymer conjugate that ensures both membrane integration and solubility.

The successful fabrication and structural integrity of the resulting RMP were verified through a suite of characterization techniques. Western blot analysis confirmed that the engineered exosomes retained classic tetraspanin markers (CD9 and CD81) and the endosomal marker TSG101, while being devoid of cellular contaminants (Calnexin and Histone H10), indicating minimal disruption to exosomal identity during the loading and functionalization process (Figure S1A). We then systematically optimized the exosome-to-PLL ratio, which revealed a correlation between PLL content and particle size. A mass ratio of 1 : 2 was selected as it achieved effective functionalization while keeping the hydrodynamic diameter under 150 nm, a critical balance for ensuring favorable *in vivo* biodistribution (Figure S1B).

With this optimal formulation defined, the structural integrity and physicochemical properties of the final RMP construct were rigorously characterized. Dynamic light scattering (DLS) quantification showed a controlled size increase from 92.89 nm for native MEX to 125.6 nm for the final construct (Figure 2B-D). Morphologically, the typical cup-shaped structure of MEX was preserved in RAPA@MEX (RM), RMP, as observed using transmission electron microscopy (TEM) (Figure 2D). The structural integrity of the exosomal membrane after drug loading and DSPE-PEG-PLL insertion was verified by cryo-transmission electron microscopy (cryo-TEM) (Figure S1C). This modest size shift was accompanied by a dramatic reversal of zeta potential from approximately -15 mV to +10 mV (Figure 2E), providing direct and compelling evidence of the successful formation of a dense, positively charged PLL corona. Confocal laser scanning microscopy (CLSM) images demonstrated strong colocalization of the RAPA (blue), MEX membrane (red), and DSPE-PEG-PLL (green) signals, further confirming the successful construction of the RMP nanocomplex (Figure 2G).

Beyond structural validation, we investigated the critical pharmaceutical properties underscoring the platform’s therapeutic potential. RMP exhibited exceptional stability in physiological buffer (PBS, 37 °C), with minimal fluctuation in size and surface charge over time, a prerequisite for systemic administration (Figure 2F). Crucially, the drug release profile was found to be intelligently responsive to the pathological microenvironment. At the acidic pH (6.0) characteristic of inflammatory sites, RMP displayed a rapid and substantial (84% within 12 h), whereas release was much more sustained at physiological pH (7.4) (Figure 2H). This “mart” pH-sensitive behavior, coupled with the protective effect of PEG layer, underscores the platform’s sophisticated design for targeted, on-demand drug delivery at disease sites, thereby maximizing local efficacy while minimizing systemic exposure and associated off-target effects.

### Engineered exosomes effectively inhibited CpG-induced inflammation

**Figure 3:**
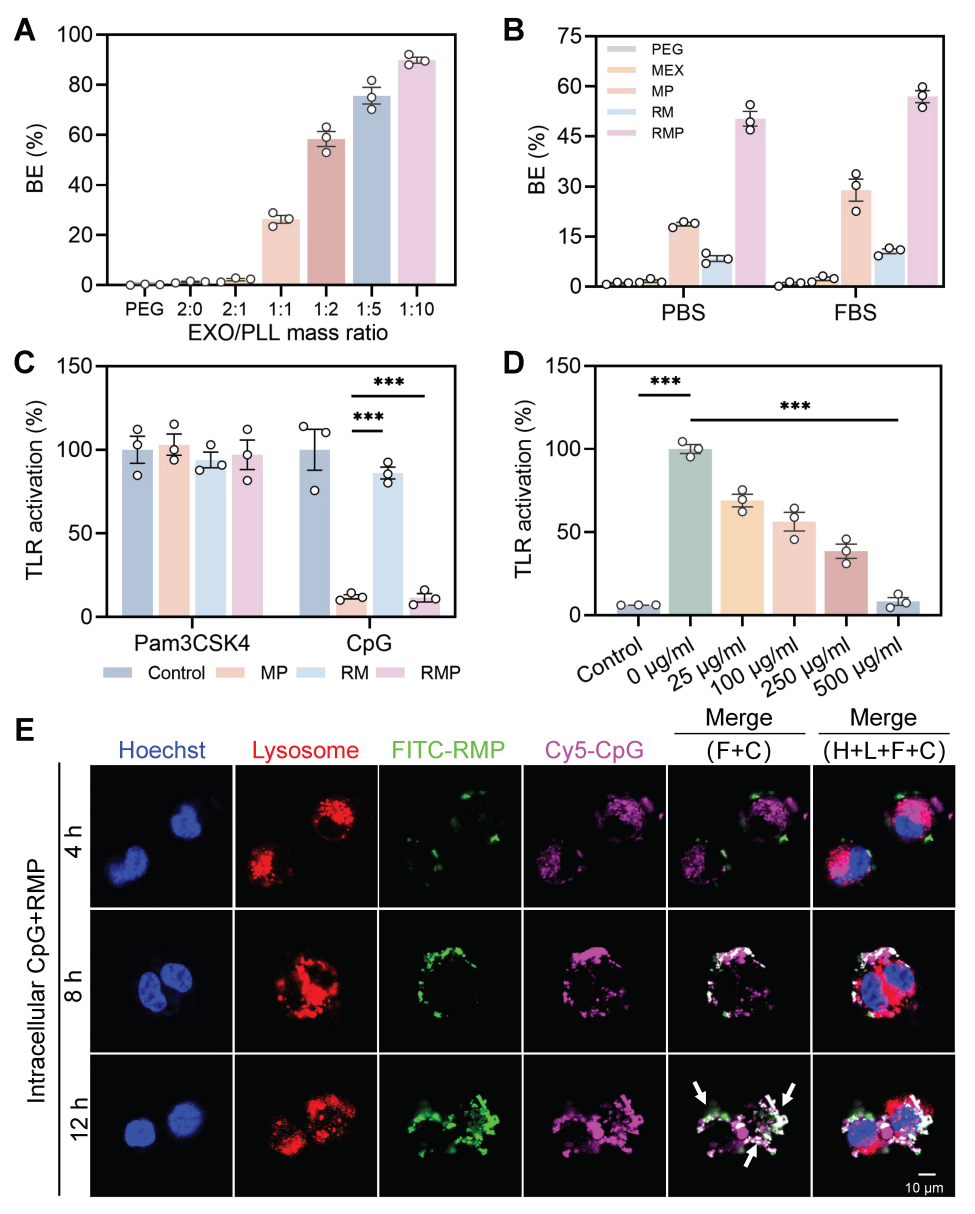
** The cationic exosome platform RMP acts as a potent cfDNA decoy to suppress TLR9 activation through dual extracellular and intracellular mechanisms.** (A) The DNA binding efficiency for exosomes and PLL at different mass ratios (mean ± SD, n = 3). (B) Quantitative analysis of cfDNA binding efficiency for the RMP complex, its components (MP and RM), and control treatments (PEG and MEX) under physiological (PBS) and serum-containing (10% FBS) conditions (mean ± SD, n = 3). (C) RMP effectively downregulate TLR9 activation driven by the CpG-ODN2006 agonist in Ramos Blue^TM^ cells (mean ± SD, n = 3). (D) Dose-dependent suppression of TLR9 signaling by RMP (mean ± SD, n = 3). (E) Analysis of time-lapse intracellular transport revealed that Quasar 670-CpG and FITC-RMP colocalize in RAW264.7 macrophages. The white arrows highlight colocalized puncta confined to endolysosomal compartments. H: Hoechst (nucleus); L: LysoTracker (lysosomes); F: FITC-RMP; C: Quasar 670-CpG. Scale bar, 10 μm. A *p*-value of < 0.05 was considered statistically significant (***p < 0.001).

The cationic surface charge of our engineered exosomes predicates their function as effective scavengers of negatively charged cfDNA. An ethidium bromide (EtBr) competitive binding assay was first employed to quantify this interaction. A positive correlation between DNA binding efficiency (BE) and the PLL-to-exosome ratio was exhibited, which can be directly attributed to the increasing density of cationic charges on the exosomal surface that enhance electrostatic interactions with negatively charged cfDNA. Based on this principle and prior optimization (Figure S1B), a mass ratio of 1 : 2 was selected for all subsequent experiments, yielding a BE of approximately 60% (Figure 3A). The lead RMP formulation exhibited superior DNA capture capability compared to its components, MEX-PL (MP) and RM. This enhanced binding affinity was evident under both physiologically mimetic conditions—Phosphate Saline Buffer (PBS, pH 7.4) and a serum-rich environment (10% Fetal bovine serum, FBS)—highlighting its potential robustness *in vivo* (Figure 3B). However, the neutral polymer (PEG) and exosome alone groups had almost no cfDNA scavenging ability. This potent DNA-binding capacity was further visually confirmed by agarose gel electrophoresis. The intensity of the CpG-ODN2006 (a cfDNA mimic) band was markedly diminished following incubation with RMP, and nearly absent compared to the MP or RM groups (Figure S2).

To validate the biological relevance of cfDNA neutralization, we investigated whether engineered exosomes could inhibit Toll-like receptor (TLR) activation in Ramos Blue™ TLR9 cells. Our findings demonstrated showed that both MP and RMP, with their positive surface charge, significantly suppressed TLR9 activation triggered by the CpG-ODN2006 agonist, while exerting no effect on non-nucleic acid activator-medicated TLR stimulation (triacylated lipopeptide Pam3CSK4) (Figure 3C). A strong dose-dependent inhibition was observed for RMP, which nearly completely suppressed TLR9 signaling at 500 μg/mL and still achieved 40% inhibition at a low concentration of 25 μg/mL, underscoring its exceptional potency as a TLR9 antagonist (Figure 3D).

As an initial step toward uncovering the mechanism of action, we first investigated whether RMP prevents the initial internalization of cfDNA. The cellular localization of FITC-conjugated RMP and Quasar 670-labeled CpG-ODN2006 in RAW264.7 macrophages was assessed by confocal laser scanning microscopy (CLSM) following 12 h of co-incubation (Figure S3A), with imaging time points at 4, 8, and 12 h. The introduction of RMP significantly reduced the intracellular uptake of cfDNA (Figure S3B), indicating that the engineered exosomes function primarily as an extracellular decoy, recognizing and sequestering pro-inflammatory cfDNA in the extracellular space and preventing its internalization.

However, a more therapeutically challenging scenario involves pathogenic cfDNA that has already been internalized by immune cells prior to treatment. To mimic this, Ramos-Blue reporter cells were subjected to a 4-hour pre-incubation with CpG, prior to the removal of free CpG and subsequent treatment with RMP. Lysosomal uptake of CpG within 4 h confirmed initial TLR9 activation (Figure S4). Strikingly, live-cell tracking demonstrated that the administered RMP was rapidly internalized and trafficked to endolysosomal compartments. Over 12 h, it progressively co-localized with the pre-loaded CpG, forming distinct punctate fluorescence signals (Figure 3E, Figure S4B). The time-dependent increase in co-localization suggests a dynamic intracellular interception capability, wherein RMP engages with pathogenic nucleic acids after their cellular uptake to disrupt ongoing TLR9 signaling. These findings, operating across cellular boundaries, demonstrate that RMP functions as a “smart immune decoy”. Its cationic shield neutralizes cfDNA extracellularly, while its native exosomal trafficking capability enables intracellular interception of cfDNA within endolysosomal compartments—the core inflammatory signaling hub.

### RAPA@MEX-PL reprograms macrophage phenotype via the mTORC1/autophagy pathway

Having established the platform’s capacity to neutralize extracellular and intracellular cfDNA, we next evaluated its direct immunomodulatory impact on macrophages, key effector cells in LN. Prior to therapeutic efficacy assessment, the biosafety of engineered exosomes was systematically evaluated. When the concentration of RAPA in RMP reached 10 µg/mL, there was no significant effect on the proliferation of macrophages, thereby defining a therapeutic window (Figure S5A). Live/dead cell staining further confirmed its biocompatibility (Figure S5B).

**Figure 4:**
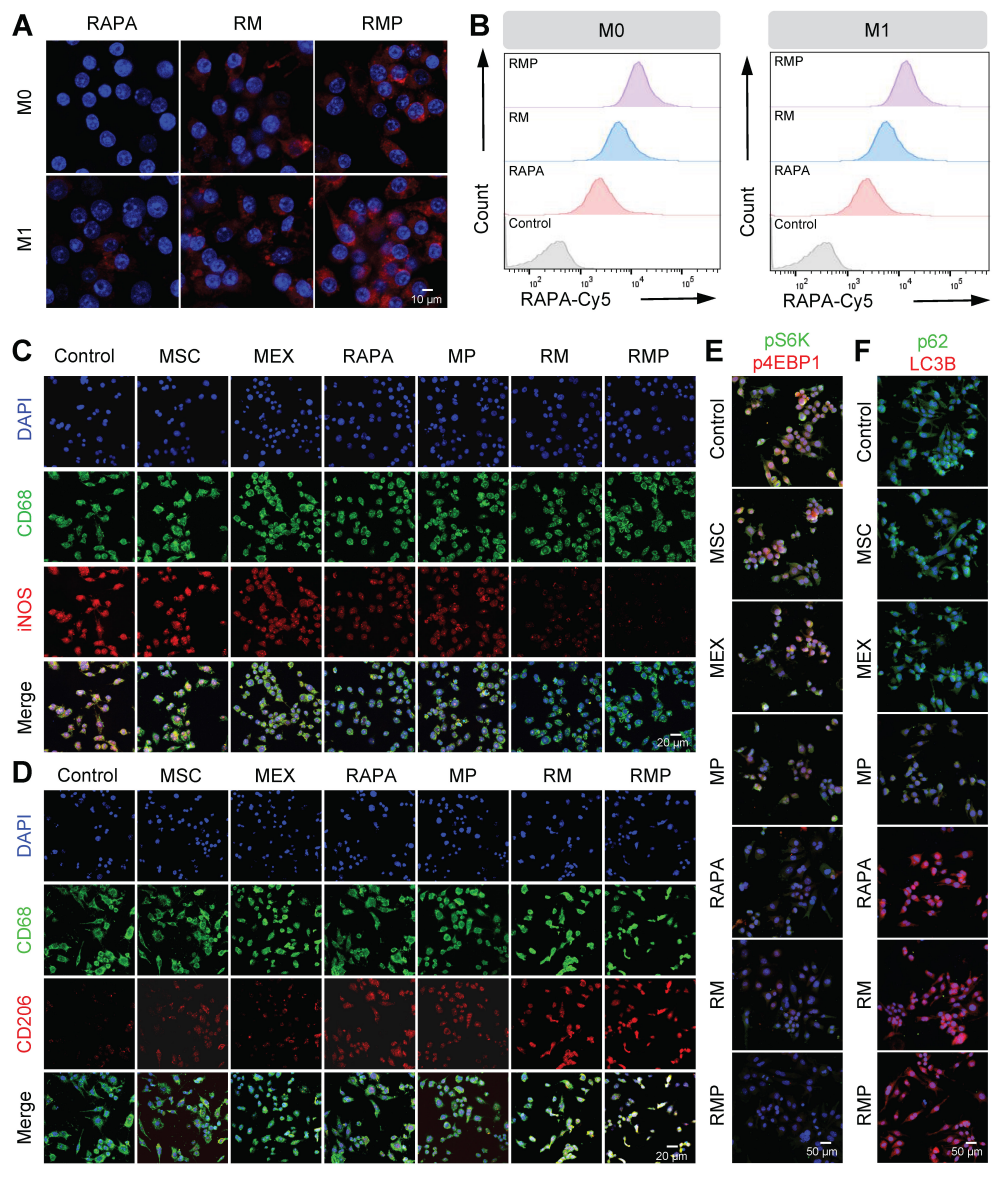
** RMP promotes M2 macrophage repolarization via enhanced cellular uptake and orchestrated modulation of the mTORC1/autophagy pathway.** (A) Confocal fluorescence microscopy images showing the cellular uptake of RAPA, RM and RMP by RAW264.7 macrophages after 1-hour incubation. Scale bar, 10 μm. (B) Quantitative flow cytometric assessment of exosome uptake corresponding to (A). (C) and (D) Representative immunofluorescence images of macrophage polarization. Macrophages were stained for CD68 (green, universal macrophage marker), iNOS (red, M1) and CD206 (red, M2), nuclei were DAPI-stained. Scale bar, 20 μm. (E) and (F) Representative immunofluorescence images of mTOR signaling and autophagic activity. Cells were stained for pS6K and p62 (green), p4EBP1 and LC3B (red), nuclei were DAPI-stained. Scale bar, 50 μm.

We then assessed the targeting and uptake efficiency in inflammatory macrophages that were incubated with Cy5-labeled RMP for 1 h. Both confocal microscopy and flow cytometry revealed that RMP was internalized more efficiently than the controls, with the RMP group showing the strongest fluorescence signal (Figure 4A-B, Figure S6A-B). This enhanced cellular uptake under inflammatory conditions is critical for delivering its immunomodulatory cargo.

Given the efficient cellular uptake and favorable biosafety, we investigated the immunomodulatory function of RMP in macrophage polarization. Under inflammatory conditions, RMP treatment significantly upregulated the M2 marker CD206 while downregulating the M1 marker iNOS, as confirmed by immunofluorescence staining (Figure 4C-D, Figure S7A-C) and flow cytometry (Figure S7D-F). In contrast, treatment with exosomes alone, PLL alone or RAPA alone exhibited significantly weaker effects on macrophage polarization compared to RMP. These findings demonstrate that the RMP platform possesses a robust capacity to remodel the dysregulated immune microenvironment, highlighting its superiority as an immunomodulatory agent.

To delineate the underlying mechanism, we probed the mTORC1/autophagy axis-a central pathway in immune cell homeostasis. As expected, mTORC1 activity was directly suppressed by all treatments containing rapamycin (RAPA monotherapy, RM, and RMP), which was confirmed by the dephosphorylation of its downstream effectors S6K and 4E-BP1 (Figure 4E, Figure S8A-C). Concurrently, these treatments activated autophagy, as evidenced by the increased LC3B levels and reduced p62 accumulation (Figure 4F, Figure S9A-C, Figure S10). Notably, the MP group, which lacks rapamycin, also exhibited a significant modulatory effect. This can be attributed to a synergistic combination of the PLL shield scavenging cfDNA to remove the pro-inflammatory trigger, and the inherent immunomodulatory molecules (e.g. regulatory miRNAs and cytokines) delivered by the MEX core itself, which collectively contribute to suppressing mTORC1 activation and promoting an anti-inflammatory state.

The superior efficacy of the complete RMP construct stems from a deliberate tripartite synergy: the polylysine shield primes the microenvironment by scavenging cfDNA to attenuate pro-inflammatory TLR9 signaling; the MEX core delivers inherent immunomodulatory cargo to establish a foundational reparative state and the controlled release of RAPA provides a precise, potent signal to drive the M2 transcriptional program via mTORC1 inhibition and autophagy activation. This integrated approach ensures that macrophage repolarization is orchestrated at both extracellular and intracellular levels.

### Effective anti-oxidant and anti-apoptotic therapies of RAPA@MEX-PL regulate podocyte function

**Figure 5:**
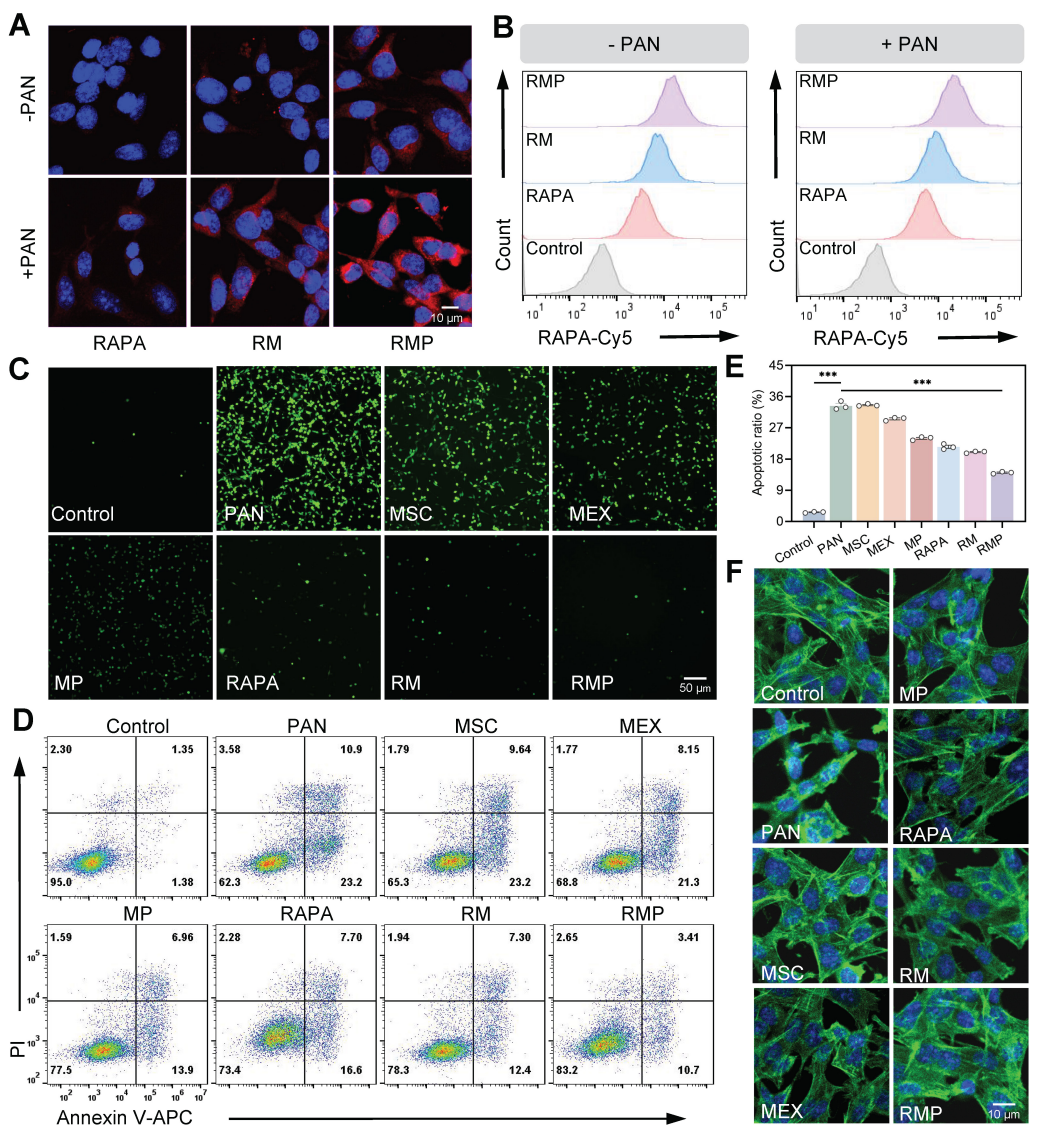
** RAPA@MEX-PL mitigates podocyte injury through integrated antioxidant and anti-apoptotic mechanisms.** (A) Confocal fluorescence microscopy images and of cellular uptake of engineered exosomes in podocytes for 1 h. Scale bar, 10 μm. (B) Quantification of engineered exosome internalization in MPC-5 cells by flow cytometry. (C) ROS levels in MPC-5 cells were assessed by fluorescence microscopy following staining with the ROS-sensitive probe DCFH-DA. Scale bar, 50 μm. (D) Flow cytometry to evaluate the anti-apoptotic role of engineered exosomes in podocytes. (E) Assessment of the anti-apoptotic effect of engineered exosomes on podocytes using flow cytometry (mean ± SD, n = 3). (F) Fluorescence images of the cytoskeleton showing F-actin distribution (phalloidin staining, green) in MPC-5. Scale bar, 10 μm. A *p*-value of < 0.05 was considered statistically significant (***p < 0.001).

The therapeutic promise of our platform extends beyond immunomodulation to direct organ protection, a critical consideration in LN where podocyte injury is a hallmark of progressive glomerular dysfunction 40-41. We first established the foundational biosafety and efficient cellular uptake of RMP in podocytes clone-5 (MPC-5), confirming its suitability for subsequent functional intervention (Figure 5A-B, Figure S11A-B, Figure S12A-C).

We next investigated the cytoprotective efficacy of RMP against puromycin aminonucleoside (PAN)-induced podocyte injury. The platform demonstrated a potent dual-action protective mechanism. First, RMP treatment significantly attenuated PAN-stimulated oxidative stress, manifested as a pronounced decrease in intracellular Reactive Oxygen Species (ROS) (Figure 5C, Figure S13A-B). Second, it concurrently, exhibited robust anti-apoptotic properties, effectively preserving cellular viability (Figure 5D-E). This coordinated mitigation of oxidative damage and prevention of cell apoptosis acted synergistically to maintain podocytes homeostasis, thereby preventing the loss of functional cells from the glomerular filtration barrier.

The functional benefits of this dual protective strategy were evidenced at the structural level. Confocal microscopy analysis demonstrated that RMP treatment effectively restored cytoskeletal architecture in PAN-stimulated MPC-5 (Figure 5F, Figure S13C), with significantly superior recovery compared to other treatment groups. The preservation of this intricate cellular skeleton is indispensable for maintaining the structural integrity of the glomerular filtration barrier.

### Spatiotemporally controlled delivery of engineered exosomes to inflamed kidney of MRL/lpr mice

While engineered exosomes have shown promising capability in recognizing and neutralizing pathogenic cfDNA under *in vitro* conditions, their targeting specificity in physiological environments requires further investigation. To examine their *in vivo* distribution, near-infrared fluorescence (NIRF) imaging was employed to track Cy5-labeled RAPA following intravenous administration in a spontaneous lupus model (MRL/lpr mice) and the health control (MRL/MPJ mice).

**Figure 6:**
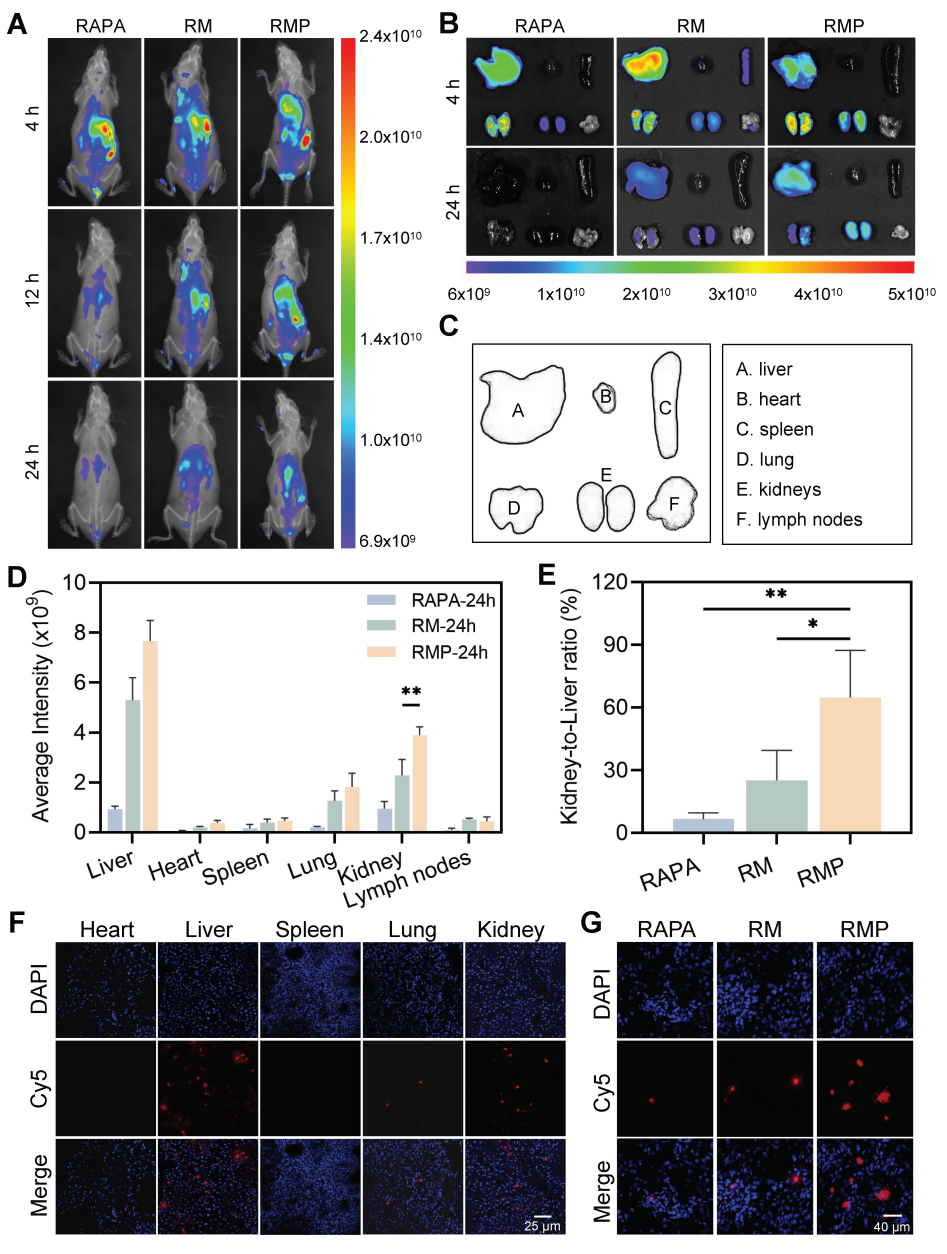
***In vivo* biodistribution of engineered exosomes in MRL/lpr mice.** (A) After intravenous injection of Cy5-labeled RAPA, RM, and RMP at designated time points, longitudinal fluorescence imaging was conducted in living mice to track their biodistribution. (B) Representative ex vivo fluorescence pictures of tissues after 4 and 24 h. (Cy5-RAPA). (C) The panel includes labels indicating the placement of each organ. (D) Quantitative analysis of Cy5 average intensity in the indicated tissues when 24 h had elapsed since the injection (mean ± SD, n = 3). (E) Kidney-to-liver ratio of Cy5 mean fluorescence intensity at 24 h post-administration (mean ± SD, n = 3). (F) Frozen sections of the major organs in RMP (Cy5-labeled RAPA) groups. Scale bar, 25 μm. (G) Confocal images of kidney cryosections from Cy5-RAPA, RM and RMP treated groups. Scale bar, 40 μm. A *p*-value of < 0.05 was considered statistically significant (**p < 0.01).

Initial whole-body imaging revealed distinct pharmacokinetic profiles (Figure 6A). Free rapamycin—known to undergo hepatic metabolism with an approximate half-life of 12 h—displayed only minimal fluorescence intensity after the injection had been given for 24 h. On the contrary, both the RM and RMP groups maintained sustained fluorescence throughout the same period, underscoring the role of the exosomal vector in prolonging systemic circulation.

Due to their lack of active targeting capability, RAPA and RM could not efficiently accumulate in the kidney, resulting in a relatively short retention time in the kidneys of MRL/MPJ mice. In contrast, RMP, modified with PLL on its surface, actively targeted the glomerular basement membrane, thereby significantly extending its renal retention time (Figure S14).

To further characterize tissue distribution, we conducted ex vivo imaging of major organs collected at 4 and 24 h post-administration (Figure 6B-C). Quantitative analysis showed that RMP achieved the highest and most persistent accumulation in the liver and kidneys (Figure 6D), suggesting a charge-dependent affinity for inflamed tissues, particularly the glomerular structures. The kidney-to-liver mean fluorescence intensity ratio was subsequently calculated, revealing that the RMP group exhibited a ratio of approximately 0.8, significantly surpassing the level measured in the RAPA group (Figure 6E). Furthermore, immunofluorescence analysis of major organ sections corroborated these distribution patterns, demonstrating selective and extensive localization of RMP within renal tissue, in stark contrast to the weak signals observed in the RM and RAPA groups (Figure 6F-G).

To directly verify whether the high renal accumulation of RMP was attributable to its active targeting capability, we performed co-localization analysis using COL4A3 (a glomerular basement membrane marker) and Cy5-labeled RAPA. The results demonstrated that in kidney tissue sections from MRL/lpr mice, the RMP group exhibited significantly stronger fluorescence signals than both the RM and RAPA groups (Figure S15). In contrast, in kidney sections from normal MRL/MPJ mice, specific fluorescence signals were detectable only in the RMP group. These findings conclusively demonstrate that the efficient renal enrichment of RMP is primarily mediated by its active targeting mechanism rather than passive tissue accumulation.

Notably, despite the pronounced accumulation of RMP in the liver—likely attributable to uptake by Kupffer cells in the hepatic sinusoids—neither histopathological evaluation of liver tissue (Figure S22) nor serum liver enzyme levels (ALT/AST, Figure S23A-B) revealed any significant signs of toxicity. These findings indicate that while RMP exhibits enhanced targeted distribution, its dosage remains within a safe therapeutic window, underscoring its favorable biocompatibility.

The observed targeting specificity is likely mediated by dual mechanisms: electrostatic attraction between the engineered exosomes and the anionic glomerular basement membrane, coupled with their strong affinity for cfDNA accumulated in the inflamed renal tissue of the lupus mouse model. This successful demonstration of spatiotemporally controlled delivery validates a core premise of our design: that engineering exogenous tropism onto native biological vectors can overcome the delivery barriers that limit conventional therapies in LN.

### Engineered exosomes demonstrated therapeutic efficacy against unprovoked murine lupus progression

**Figure 7:**
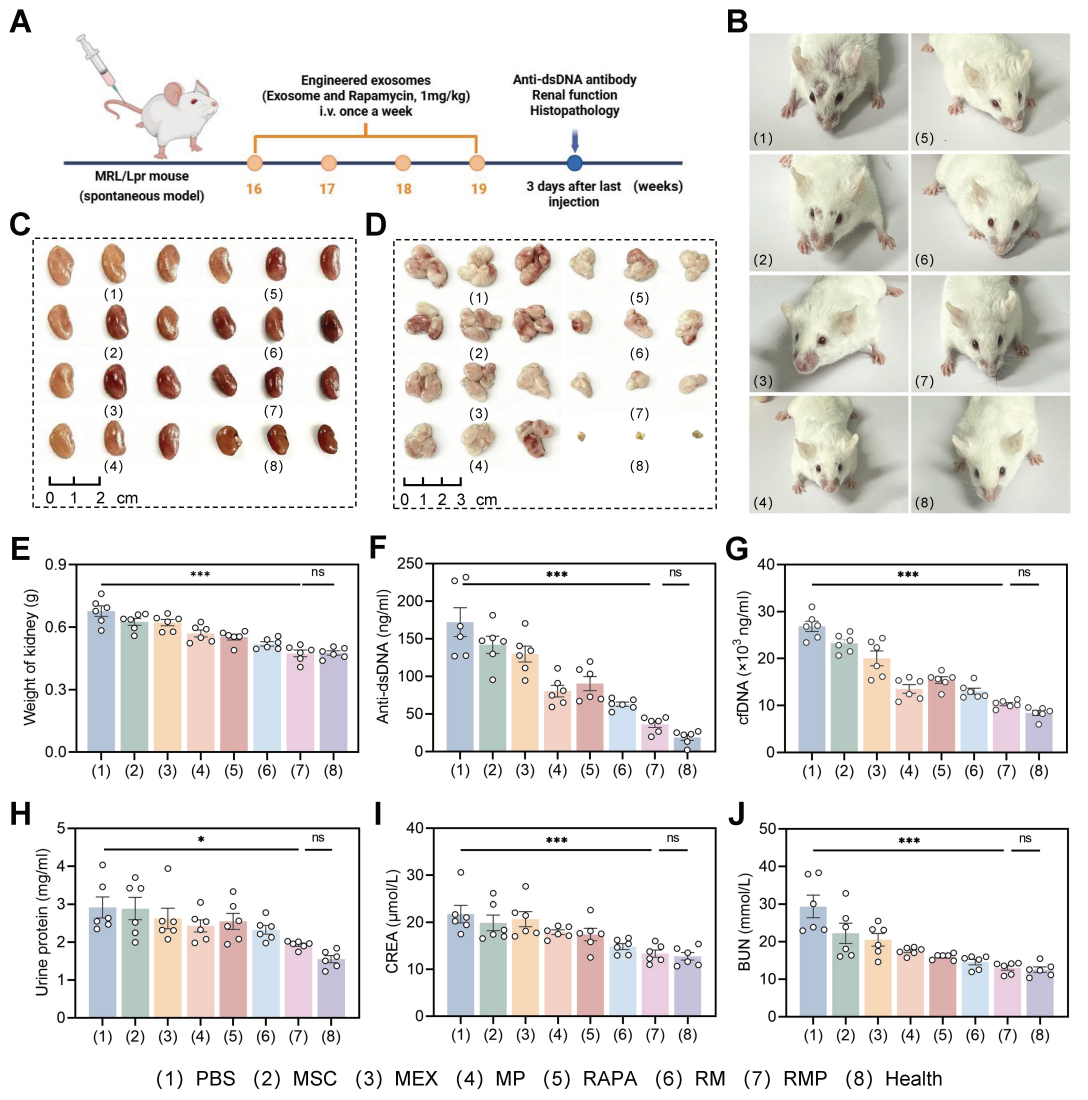
**Therapeutic efficacy of engineered exosome-based RAPA delivery in MRL/lpr mice.** (A) Schematic diagram illustrating the therapeutic regimen administered to MRL/lpr mice. (B) Typical facial photographs of lupus-prone mice from the indicated treatment groups captured after the treatment period. (C) and (D) Representative pictures of isolated kidney (C) and lymph gland (D) following the various treatments. (E) Weight of kidney after different treatments (mean ± SD, n = 6). (F) Serum dsDNA and (G) cfDNA concentrations in LN mice from all eight groups at week 19 (mean ± SD, n = 6). (H-J) Urinary protein, creatinine and urea nitrogen levels in lupus mice from the indicated groups at week 19 (mean ± SD, n = 6). A *p*-value of < 0.05 was considered statistically significant (*p < 0.05 and ***p < 0.001).

The multi-component platform not only demonstrated potent therapeutic efficacy *in vitro* by clearing cfDNA, modulating immunity, and protecting organs, but also demonstrated a pronounced targeting capacity toward cfDNA accumulated within the inflamed renal tissue of lupus-prone mice. Therefore, assessment of the *in vivo* therapeutic effects was performed in MRL/lpr mice subjected to a five-week regimen of weekly tail vein injections. The study included a comprehensive set of controls: disease controls (PBS), parental cell therapy (MSC), native exosomes (MEX), cationic exosomes (MP), free low-dose drug (RAPA), drug-loaded exosomes (RM), and our lead formulation (RMP), alongside a baseline healthy (MRL/MPJ) group (Figure 7A). Notably, the RAPA dosage utilized in this study was significantly lower than those conventionally employed in monotherapy. No substantial body weight changes were observed across groups during the treatment period (Figure S16A), indicating favorable treatment tolerance.

Therapeutic outcomes were significantly superior in the RMP group, which exhibited more substantial improvement in facial skin lesions (Figure 7B) and more pronounced reduction in organ weights than any other treatment group (Figure 7C-E, Figure S16B-D). These data indicate that the combinatorial strategy of RAPA and engineered exosomes produces synergistic efficacy, underpinning more effective disease management. In contrast, the PBS, MSC, MEX and RAPA monotherapy groups showed limited therapeutic efficacy, likely attributable to inadequate tissue targeting and rapid systemic clearance.

At the serological level, RMP-treated mice demonstrated substantially reduced anti-dsDNA antibody levels (Figure 7F), a key biomarker of disease activity, indicating effective suppression of autoimmune responses. This was accompanied by a pronounced clearance of serum cfDNA (Figure 7G), an effect most evident in the groups containing the cationic polylysine modification (MP and RMP), suggesting that cationic modification improves nucleic acid binding affinity and prolongs retention time—critical factors for neutralizing pathogenic cfDNA. This serological improvement highlights a key mechanistic hierarchy: while the cationic shield is necessary and sufficient for effective cfDNA scavenging, it is the full RMP construct—integrating cfDNA clearance (PLL), innate immunomodulation (MEX), and mTOR inhibition (RAPA)—that is required to fully suppress the downstream adaptive autoimmune response (anti-dsDNA).

Most importantly, RMP treatment conferred profound renal protection. At week 19, the RMP group exhibited near-normal renal function. Urinary protein excretion was low. Serum creatinine and blood urea nitrogen (BUN) likewise approached the levels seen in healthy MRL/MPJ controls (Figure 7H-J). This restoration of near-normal renal function demonstrates that our multi-component platform successfully translates systemic immunomodulation into concrete organ protection, addressing the ultimate clinical failure in LN—end-stage renal disease. In contrast, the modest effects in PBS, MSC, MEX, and RAPA control groups emphasize the necessity of our combinatory strategy for the concurrent resolution of dual therapeutic barriers: inadequate targeting and a singular mode of action.

**Figure 8:**
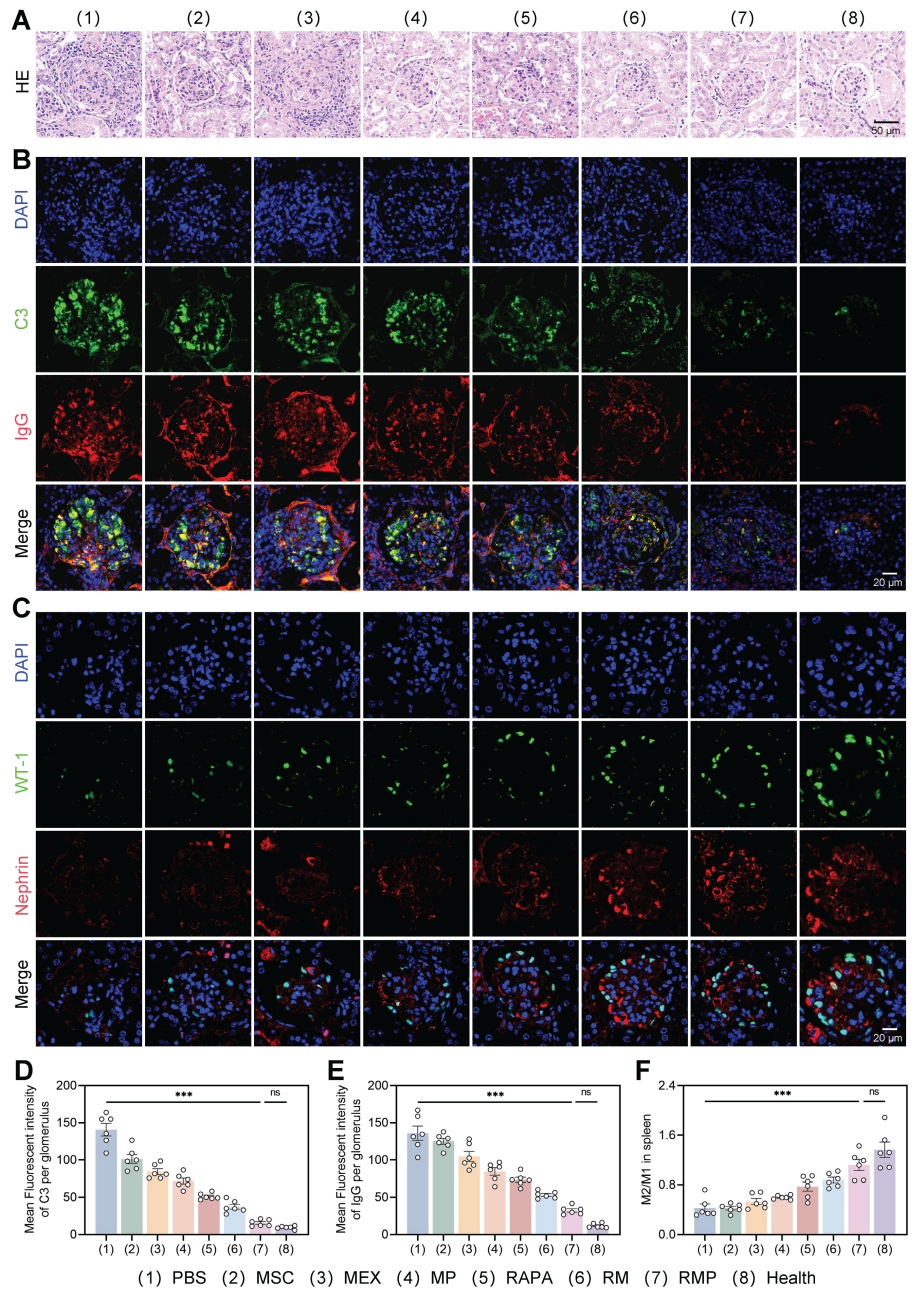
**Engineered exosomes attenuated lupus nephritis in MRL/lpr mice.** (A) H&E staining of renal tissue across the eight experimental groups (representative images). Scale bar, 50 μm. (B) Renal tissue sections stained for C3 (green) and IgG (red) by immunofluorescence. DAPI counterstaining (blue) labeled cell nuclei. Scale bar, 20 μm. (C) Immunofluorescent staining of WT-1 (green) and nephrin (red). DAPI counterstaining (blue) labeled cell nuclei. Scale bar, 20 μm. (D) and (E) Statistical plots of the mean fluorescence intensity of C3 and IgG in the positive areas of individual glomeruli (mean ± SD, n = 6). (F) Statistical analysis of the M2/M1 macrophage ratio in the spleens of lupus mice (mean ± SD, n = 6). A *p*-value of < 0.05 was considered statistically significant (***p < 0.001).

The superior therapeutic outcomes observed at the functional level were corroborated by comprehensive histological and immunological analyses. Hematoxylin-eosin (H&E) staining of renal sections demonstrated that relative to the PBS group, the MP, RM, and RMP groups exhibited significantly reduced glomerular hypertrophy, along with decreased periglomerular inflammatory cell infiltration and attenuated glomerulonephritic damage (Figure 8A). Immunofluorescence detection of C3 and IgG deposition in the glomeruli showed that, compared with the PBS group, all other groups had reduced deposition of C3 and IgG, with the RM and RMP groups showing a significant decrease (Figure 8B). Effective suppression of immune complex (IC) accumulation was observed in the RMP group, where deposition levels were equivalent to those of healthy controls (Figure 8D-E).

We further assessed the status of glomerular podocytes via immunofluorescence. First, podocyte counts were evaluated using the nuclear marker WT-1, which revealed a significant increase in podocyte numbers in both the RM and RMP groups as opposed to untreated model group. Second, the expression of nephrin, a key protein of the slit diaphragm, was examined to assess podocyte functional integrity, and a marked upregulation of nephrin fluorescence intensity was observed in these two groups (Figure 8C). Notably, both key parameters measured in the RMP group were restored to levels approaching those seen in the healthy control group, indicating that this intervention not only effectively increased podocyte numbers but also promoted the repair of podocyte foot process structure and filtration barrier function.

To systematically evaluate the immunomodulatory capacity of the multi-component platform and elucidate its role in reestablishing immune homeostasis, splenic macrophage polarization was assessed. Treatment with RAPA, RM, and RMP significantly skewed macrophages toward an M2-dominant phenotype, with RMP producing the highest M2/M1 ratio (Figure 8E, Figure S17). This cellular-level transition to an anti-inflammatory state was further supported by cytokine data, which indicated a marked upregulation of anti-inflammatory cytokines alongside downregulation of pro-inflammatory counterparts in all experimental groups versus the PBS control, with the RMP group demonstrating the most favorable immunologic rebalancing (Figure S18A-B). Collectively, these findings substantiate that cfDNA clearance, innate immune regulation, and mTOR inhibition act synergistically to promote anti-inflammatory macrophage polarization. This coordinated approach proves effective in driving a systemic shift toward an anti-inflammatory state, underscoring its therapeutic potential for autoimmune pathologies such as lupus nephritis.

To further evaluate whether the engineered exosome RMP possesses the potential for enhanced efficacy and reduced toxicity compared to rapamycin monotherapy in the treatment of lupus-prone mice, this study included a standard-dose rapamycin group (Rapa-Standard, Rapa-S) and a high-dose rapamycin group (Rapa-High, Rapa-H) as controls. After four weeks of administration via tail vein injection, body weight in the Rapa-H group decreased progressively over time, relative to the low-dose rapamycin group, both the Rapa-S and Rapa-H groups exhibited greater improvements in facial skin lesions, kidney weight, lymph node weight, spleen weight, as well as in serological and urinary parameters (Figure S20). Furthermore, in renal pathological sections, H&E staining (Figure S21A), immunofluorescence staining for IgG and C3 (Figure S21B), and immunofluorescence staining for nephrin and WT-1 (Figure S21C), the Rapa-S and Rapa-H groups also demonstrated more pronounced amelioration of lupus nephritis; however, their therapeutic effects were still inferior to those of the RMP engineered exosome group (Figure S21D-G).

Subsequent toxicological evaluation was conducted through H&E staining of major organs and liver function analysis. The results revealed that in the Rapa-S and Rapa-H groups, H&E staining of liver tissue showed marked sinusoidal dilation, congestion, and the presence of blood-filled lacunae. In the Rapa-H group, hepatocellular steatosis was also observed (Figure S22). Additionally, the Rapa-S group displayed rising trends in serum ALT and AST, while those in the Rapa-H group exceeded the normal range, indicating potential liver injury. In contrast, all parameters and histomorphological findings in the other groups were within normal ranges (Figure S23A-D), further confirming their favorable biocompatibility.

These findings collectively demonstrate the superior efficacy of the RMP hybrid system, which strategically integrates RAPA, MEX and PLL to synergistically control SLE progression and alleviate nephritis. This multi-component design leverages their respective strengths: First, the surface PLL efficiently clears cfDNA at the systemic circulation level, reducing the formation and deposition of immune complexes at the source and mitigating the initiating factors of LN. Second, upon accumulation in the kidney, the MSC-derived exosomes carried by RMP, along with the released RAPA, jointly act on infiltrating macrophages. By inhibiting mTOR and inducing autophagy, they promote macrophage repolarization toward the M2 phenotype. This exerts a dual effect at the tissue microenvironment level: On the one hand, M2 macrophages secrete anti-inflammatory factors, further alleviating inflammation; on the other hand, they enhance the phagocytic clearance of deposited IC, which echoes the first mechanism. Finally, directly targeting the key terminal cells in LN-podocytes, RAPA and MEX protect glomerular podocytes by inhibiting oxidative stress, thereby preserving renal function. Thus, these three mechanisms are not isolated; rather, they form a multidimensional and mutually reinforcing synergistic therapeutic network, operating from the circulatory system to renal tissue and down to specific cells.

## Conclusion

In summary, this study developed an engineered exosome-based multifunctional nanoplatform with dual functions of clearance of circulating cfDNA and targeted delivery of RAPA for the treatment of LN. By combining exosomes, RAPA and PLL, we successfully constructed three composite systems, including MP, RM and RMP, and systematically evaluated their biocompatibility, DNA clearance ability and TLR inhibition effect. This “smart immune decoy” platform leverages a synergistic triad of functions: the cationic PLL shield for effective cfDNA scavenging and renal targeting, the MSC-derived exosome core for innate immunomodulation, and the controlled release of rapamycin for localized mTOR inhibition. This multi-component design enabled a comprehensive therapeutic effect, demonstrated by the potent inhibition of TLR9 signaling, the reprogramming of macrophages toward an M2 phenotype, and direct podocyte protection *in vitro*, culminating in significantly attenuated systemic autoimmunity and renal injury in a spontaneous lupus model.

Our work introduces a novel “smart immune decoy” platform (RMP) that uniquely integrates cfDNA scavenging, systemic immunomodulation, and direct renal protection. Its design advances the field through three key innovations: 1) The PLL corona functions both as an electrostatic sponge for neutralizing systemic cfDNA and as a kidney-targeting moiety via charge interaction with the anionic glomerular basement membrane; 2) Beyond promoting M2 macrophage repolarization via mTOR inhibition, the engineered exosomes directly deliver RAPA to renal cells, protecting podocytes from oxidative stress and apoptosis; 3) The platform establishes a spatiotemporal therapeutic network that concurrently disrupts cfDNA-driven inflammation, reshapes the immune microenvironment, and preserves the glomerular filtration barrier. Thus, this platform constitutes a multi-layered therapeutic system tailored to the complex pathophysiology of LN. By integrating the biocompatibility and intrinsic bioactivity of natural exosomes with the precision functionality of synthetic engineering, it will open new avenues for the development of next-generation targeted immunotherapies.

Although RMP shows favorable targeting and efficacy in short-term treatment, the chronic nature of lupus nephritis calls for consideration of its long-term safety. Repeated administration of PLL and PEG may induce immune responses and trigger the accelerated blood clearance (ABC) phenomenon, compromising subsequent efficacy. Addressing these immunogenicity issues is critical for clinical translation. Future work will focus on low-immunogenicity stealth polymers, such as polyglycerol and poly (oxazoline), to facilitate clinical advancement 42.

## Supplementary Material

Supplementary materials and methods, figures.

## Data Availability

The data on which the results of this study are based are available in the supplementary material of this article.

## Abbreviations

SLE: Systemic lupus erythematosus
LN: lupus erythematosus
cfDNA: cell-free DNA
MEX: mesenchymal stem cell-derived exosomes
PLL: polylysine peptides
ROS: reactive oxygen species
LPS: lipopolysaccharide
IFN-γ: interferon-gamma
Pam3CSK4: Pam3Cys-Ser-(Lys)4
PI: propidium iodide
DCHF-DA: 2’, 7’-Dichlorodihydrofluorescein diacetate
PAN: puromycin aminonucleoside
DSPE-PEG-PLL: 1, 2-Distearoyl-sn-glycero-3-Phosphoethanolamine-Polyethylene-Glycol-2000-Poly-L-lysine
NIRF: near-infrared fluorescence
DSPE-PEG-FITC: 1, 2-distearoyl-sn-glycero-3-phosphoethanolamine-N-[Fluorescein Isothiocyanate (polyethylene glycol)-2000]
MPC-5: podocyte clone-5
H&E: hematoxylin-eosin
