# Supplementary material for: Exosomal immune decoy integrates cfDNA scavenging and mTOR inhibition for synergistic lupus nephritis therapy
Source: Theranostics. 2026 May 11;16(12):6763–82. doi: 10.7150/thno.130906 (PMC13231985; doi:10.7150/thno.130906)
Supplement: Supplementary file 1 — Supplementary materials and methods, figures. [file thnov16p6763s1.pdf]

Supplementary Information

Exosomal immune decoy integrates cfDNA scavenging and mTOR inhibition for synergistic lupus nephritis therapy

Mingxin Zhu[1,2], Qinyao Xu[2], Zhicheng Tang[1,2], Zexin Wang[2], Haofang Zhu[2,*], Lingyun Sun[1,2,3,*]

1. Department of Rheumatology and Immunology, The Second Affiliated Hospital of Anhui Medical University, Hefei, China

2. Department of Rheumatology and Immunology, The First Affiliated Hospital of Anhui Medical University, Hefei, China

3. Department of Rheumatology and Immunology, Nanjing Drum Tower Hospital, Affiliated Hospital of Medical School, Nanjing University, Nanjing, China

Correspondence: hfzhu@ahmu.edu.cn, lingyunsun@nju.edu.cn

Materials and Methods

Material

DSPE-PEG-PLL, Cy5-rapamycin, and DSPE-PEG-FITC were ordered from Ruixi Biotechnology Co., Ltd (Xian, China). DMEM/F12 and fetal bovine serum (FBS) were ordered from Gibco. Phosphate-buffered saline (PBS, pH 7.4) and DMEM were commercially available from the Servicebio Biology Co., Ltd (Wuhan, China). Iscove's Modified Dulbecco's was ordered from Thermo Fisher Scientific (USA). CpG 2006 and its Cy5.5-labeled counterpart were sourced from Genscript China. A 100 μg/mL stock solution of unlabeled CpG was prepared by diluting the compound in PBS. The Annexin V-APC apoptosis kit was acquired from Elabscience. The Bradford Protein Content Assay Kit was sourced from Beyotime Biotechnology Co., Ltd. Additionally, the CCK-8 assay kit, Calcein AM, propidium iodide (PI), Actin-Tracker Green (a microfilament-specific dye), Lysotracker Red DNA-99, 2’, 7’-Dichlorodihydrofluorescein diacetate (DCHF-DA) (for ROS detection), and anti-fade mounting medium with DAPI were all procured from Beyotime Biotechnology Co., Ltd. Rapamycin (RAPA, 10 mM*1 mL in DMSO), calf thymus DNA, Polyethylene glycol 35000 (PEG), puromycin aminonucleoside (PAN), Pam3Cys-Ser-(Lys) 4 (Pam3CSK4), lipopolysaccharide (LPS) and Interferon-gamma (IFN-γ) were obtained from MedChemExpress. Antibodies used for western blot, including CD9, CD81, TSG101, Alix, Histone H10, and Calnexin, were ordered from Abclonal Biological Technology Co., Ltd (Wuhan, China). Antibodies used for flow cytometric analysis were as follows: for in vitro experiments, FITC-CD86 and APC-CD206; for in vivo experiments, a panel including F4/80-PE, CD86-PE/Cy7, CD11B-FITC, CD206-APC and LY-6G-PerCP/Cy5.5. All antibodies were obtained from BioLegend (USA). Antibodies used for laser confocal microscopy analysis, including anti-CD68, anti-CD206, and anti-iNOS, were obtained from Invitrogen. Antibodies used for immunofluorescence analysis, including pS6K p62 and COL4A3 were obtained from Proteintech, p4EBP and LC3B were obtained from Zenbio. Anti-dsDNA antibody levels (FineTest), enzyme-linked immunosorbent assays

(MultiSciences).

Cells and culture of primary cells

MSCs were supplied by Taisheng Corp (Nanjing) and cultivated in DMEM/F-12 complete medium. Mouse podocyte clone-5 (MPC-5) was obtained from the Servicebio (Wuhan) and incubated in RPMI 1640 media. RAW264.7 was obtained from the Servicebio (Wuhan) and incubated in DMEM high glucose media. Invitrogen (USA) was the source of Ramos Blue™ reporter cells cultured in IMDM complete medium (serum inactivated). The authenticity and functionality of these cells have been confirmed through consistent use in multiple laboratories.

Isolation and identification of MEX

MSCs at passages 3-8 were first cultured in serum-containing medium. When the cell density reached 80%, the cells were washed twice with PBS following medium removal, and then the culture was switched to serum-free medium. After 3 days of culture, the cell supernatant was harvested and cleared by sequential centrifugation at 4 °C with progressively increasing forces: 300 g (15 min), 2,000 g (20 min), and 10,000 g (30 min). After each centrifugation step, the supernatant was retained, and the pellet was discarded. Concentration of the resulting supernatant was carried out using a 100-kDa molecular weight cut-off (MWCO) ultrafiltration tube (Merck Millipore). To isolate exosomes, the concentrated sample was subjected to centrifugation (12,000 g, 2 h, 4 °C). The recovered exosomes were then resuspended in PBS. The protein content of the exosome preparation was quantified using a protein assay kit, while Dynamic light scattering (DLS) was used to determine particle size and particle number (Zimeng Technology Co., Ltd., China) (About 15 μg exosomes were extracted from 100 ml of serum-free MSC cell supernatant, and the concentration of exosome Particles was 2.5E+9 Particles/mL. The ratio of protein to particle number was 6 μg/10^9). The morphological structure of exosomes was observed by transmission electron microscope (TEM, FEI Tecnai G2 Spirit, USA). The membrane structure of exosomes was observed by cryo-transmission electron microscopy (cyto-TEM, FEI Talos F200C). The expression of CD9, CD81, Alix, Histone H10, and Calnexin on MEX was analyzed by Western blot.

Synthesis and Characterization of RAPA@MEX-PL

Rapamycin was mixed with MEX (Rapamycin : MEX = 1 : 1, mass ratio) for incubating 10 s. The mixture was then subjected to ultrasonication using a probe-type cell crusher (Xiaomei Ultrasonic Instrument Co., Ltd.). The ultrasonication was performed at 25% of the maximum power output and a frequency of 25 kHz, operating in pulsed mode with 6 cycles of 30 s on and 30 s off. The whole process was conducted in an ice bath to avoid heat-induced damage, subsequently maintained at 37 °C over a 1-hour period to allow membrane recovery. Ultrafiltration centrifugation was used to remove the free RAPA. DSPE-PEG$_{2k}$-PLL was dissolved in 10 mM HEPES at 55 ℃ for 15 s. The resulting suspension was mixed with RAPA@MEX solution for 1 h at 37 ℃ (DSPE-PEG$_{2k}$-PLL : MEX = 2 : 1, mass ratio) to obtain RAPA@MEX-PL. The product was purified using a 1000 kDa ultrafiltration tube. Subsequent characterization included assessment of morphology, particle size distribution, and zeta potential. These analyses were performed by TEM and DLS.

Quantification of encapsulation efficiency and drug loading capacity

Rapamycin absorbance at 278 nm was recorded on a microplate reader for quantification of drug loading and encapsulation performance. A calibration curve covering 0-50 μg/mL rapamycin was established. Encapsulation efficiency (EE%) was defined as the ratio of encapsulated rapamycin mass to the initial rapamycin mass, expressed as a percentage. Drug loading capacity (DL%) was similarly calculated as the percentage of rapamycin mass relative to the total nanoparticle mass.

Colocalization of RAPA@MEX-PL

Fluorescent labeling of RAPA, MEX and polylysine (mass ratio, 1 : 1 : 2) was performed using Cy5, DIR and FITC, respectively. Confocal microscopy (Leica, STELLARIS STED) was used to visualize the fluorescently labeled RAPA@MEX-PL.

RAPA@MEX-PL storage stability

To assess their stability over time, RAPA@MEX-PL (1 mg/ml MEX) were suspended in PBS and stored at 37 ℃ for 7 days, with periodic measurements of their average diameter and zeta potential.

The release of RAPA from RAPA@MEX-PL in vitro

To investigate the drug release profile of rapamycin, RAPA@MEX-PL or free RAPA were separately dissolved in 5 mL of PBS (pH 6.0 or 7.4). The resulting solutions were transferred into dialysis membranes rated for 100 kDa (i.e., retaining molecules above this molecular weight). Immersion of the dialysis bags was carried out using 20 mL of PBS, followed by incubation at 37 °C with orbital shaking at 80 rpm. At predetermined time points, 1 mL of the external buffer (outside the dialysis membrane) was withdrawn to measure the drug concentration using a multimode microplate reader (Thermo Fisher). After each sampling, 1 mL of fresh PBS was replenished to maintain a constant volume.

Evaluation of cfDNA-Binding Efficiency of Engineered Exosomes

Separate solutions of calf thymus DNA and ethidium bromide (EtBr) were prepared in PBS at 1 mg/mL each. A reaction mixture was prepared by combining the following components: calf thymus DNA (4 μL), EtBr (4 μL), specified volumes of RMP, RM, MEX, or PEG, and either FBS or PBS (16 μL). The final volume was adjusted to 160 μL with PBS. Incubation of the mixture proceeded at 37 °C over 24 h. The supernatant (100 μL) was then gently transferred to a new 96-well plate, and its fluorescence signal was recorded using a multimode microplate reader (Thermo Fisher; λ_{ex} = 485 nm, λ_{em} = 590 nm). The nucleic acid binding efficiency of the cationic materials was quantified according to the equation below. Binding efficiency (%) = $[(1-(A-A_0/A_1-A_0)] \times 100\%$. Fluorescence intensity was measured for three samples: (i) the EtBr/DNA complex present in the supernatant after incubation (termed A); (ii) free EtBr (termed A_0); and (iii) the untreated EtBr/DNA complex (termed A_1).

To test the cfDNA-binding efficacy

Cationic materials were first incubated with CpG (0.4 mg/mL) at 4 °C for 24 h. Bromophenol blue (0.25%) was added to the resulting mixture, which was then separated by electrophoresis on a 2% agarose gel. Electrophoresis was carried out at 120 V for 40-45 min. Gel visualization was achieved with a WB600Pro imaging system. (BLT Biotechnology Co., Ltd.).

Extracellular agonist-mediated suppression of Toll-like receptor activation

The ability of engineered exosomes to inhibit TLR9 activation was evaluated using Ramos Blue™ reporter cells. Cells were seeded in 96-well plates at a density of 5 × 10^4 cells per well. After cell attachment, Pam3CSK4 (1 μM) and CpG-ODN2006 (1 μM) were mixed with different components and then added to the 96-well plate. Following incubation at 37 °C for 24 h, the supernatant was collected. The QUANTI-Blue assay was used to assess SEAP (secreted embryonic alkaline phosphatase) activity, following the manufacturer's instructions. The supernatant was combined with the detection medium. Following a 1-hour incubation at room temperature, the absorbance was read at 650 nm using a multimode microplate reader. TLR activation was expressed as $(X-X_0)/(X_1-X_0) \times 100\%$, with X, X_0, and X_1 corresponding to the optical density of the test, control, and agonist-only groups, respectively.

Cellular colocalization of CpG and engineered exosomes

One day prior to the experiment, confocal culture dishes were seeded with RAW264.7 cells at a concentration of 2 × 10^4 cells per dish. On the following day, two parallel experiments were performed. In the first experiment, two fluorescent probes were added simultaneously: Quasar 670-conjugated CpG-2006 (1 μM) and exosomes labeled with FITC. This was done to assess whether the engineered exosomes could reduce cellular uptake of cfDNA. In the second experiment, cells were subjected to an initial 4-hour incubation. During this time, they were exposed to Quasar 670-labeled CpG-2006 at a concentration of 1 μM., followed by the addition of FITC-labeled engineered exosomes to evaluate whether the engineered exosomes could bind to intracellularly internalized CpG. At 4, 8, and 12 h time points, the incubation was terminated. Lysosomes were visualized by staining with Lyso Tracker Red DND-99. Nuclei were counterstained separately with DAPI. Imaging was performed using a confocal microscope (Leica STELLARIS STED).

The CCK-8 assay

Briefly, RAW264.7 cells were prepared for the experiment by seeding them into 96-well plates. The seeding density was 1 × 10^5 cells per well, and this was done on

the evening before the experiment. After overnight culture, the original medium was replaced with fresh complete medium containing graded concentrations of rapamycin or engineered exosomes. Cells were then incubated for a further 24 h or 48 h, after which the medium was removed and CCK-8 working solution was added for viability assessment. The absorbance of each well was recorded at 450 nm using a multimode microplate reader (Thermo Fisher).

The Calcein/PI cell cytotoxicity assay

After seeding and attachment in 24-well plates, the culture medium was replaced with complete medium containing the indicated treatments and incubated for 24 h. Subsequently, preparation of the dye solution followed the manufacturer's guidelines, after which it was kept in darkness for a 30-minute incubation. Cell images were captured using a Nikon Ti2-A microscope.

In Vitro Cellular Uptake Study

Coverslips were placed in 12-well plates, and RAW264.7 and MPC-5 cells were used for seeding. The cell density was adjusted to 1×10^6 cells per well. RAW264.7 cells were stimulated with 10 μg/mL LPS (induces M1 polarization) and 20 ng/mL IFN-γ (synergizes with LPS for M1 polarization) for 24 h. MPC-5 cells were divided into two groups. One group was stimulated with PAN (15 μg/mL), which induces podocyte injury. The other group served as untreated controls. 24 h later, they were incubated for 2 h with Cy5-labeled formulations: RAPA (10 μM), RAPA@MEX (9.55 μg/ml MEX, 10 μM RAPA), and RAPA@MEX-PL (9.55 μg/ml MEX, 10 μM RAPA). Following PBS washes, Cy5 internalization was quantified by flow cytometry (CytoFLEX, Beckman Coulter, USA). In parallel, nuclei were stained with DAPI for confocal imaging (Leica STELLARIS STED).

Macrophage Polarization

RAW264.7 cells were treated as described above to induce M1 polarization. Following a 24-hour exposure to the drug, the culture supernatant was aspirated, and the cell monolayer was gently rinsed twice with PBS. Subsequently, fixation was performed using 4% paraformaldehyde for 15 min at ambient temperature, followed by a 10-minute permeabilization step with 0.3% Triton X-100. After two additional

PBS washes, non-specific binding was blocked by incubating the cells with 5% FBS for 30 min. The cells were then incubated overnight at 4 °C with primary antibodies targeting iNOS, CD206, and CD68. On the following day, secondary antibody incubation was carried out for 1 h at room temperature. Nuclear counterstaining was achieved with DAPI. Fluorescence images were acquired using a confocal microscope. For flow cytometric analysis, cells were detached and subjected to a single PBS wash. Cells were then stained with a live/dead viability dye for 20 min, and the reaction was terminated with PBS. After centrifugation, cells were surface-stained with a prepared solution containing CD86 antibody for 20 min, followed by PBS termination and centrifugation. Cells were then fixed and permeabilized, followed by intracellular staining with a prepared solution containing CD206 antibody for 30 min. PBS was used to terminate the reaction, after which the cells were resuspended in 200 μL of the same buffer and immediately analyzed on a CytoFLEX instrument (Beckman Coulter, USA). FlowJo software (FlowJo, USA) was used for data analysis.

Assessment of mTOR and autophagy signaling pathways

RAW264.7 cells, following polarization induction and a 24 h drug exposure. Fixation of the cells was carried out with 4% paraformaldehyde for 15 min at ambient temperature. Permeabilization was subsequently achieved using 0.3% Triton X-100 over a 10-minute period. Overnight incubation at 4 °C was conducted using primary antibodies directed against pS6K and p4EBP1 (mTOR signaling) or p62 and LC3B (autophagic flux). After two washes in PBS, the samples were treated with secondary antibodies (1 h, room temperature). Nuclear counterstaining followed using DAPI. The dye contained an anti-fade reagent. Imaging was performed on a Leica STELLARIS STED confocal microscope. The expression of p62 and LC3B (autophagy flux) on RAW264.7 cells were also analyzed by Western blot.

The cell apoptosis assay

For the experiment, MPC-5 cells were prepared in 6-well plates. This cell seeding was carried out one day in advance. After cell attachment, the culture medium was supplemented with PAN at a concentration of 15 μg/mL together with engineered exosomes at indicated concentrations. Following a 24 h treatment, the apoptosis rate

was assessed. Staining was carried out as per the manufacturer's protocol. Subsequently, the samples were analyzed on a flow cytometer.

Measurement of ROS

The culture medium was first removed. Subsequently, the cells were gently rinsed with warm PBS. Preparation of 10 µmol/L DCFH-DA was performed according to the manufacturer's directions. The probe was then incubated with the cells at 37 °C for 30 min, and the cells were subsequently rinsed three times using warm buffer, and then imaged with a fluorescence microscope (Ti2-A, Nikon). The resulting fluorescence intensity was quantified using ImageJ software.

Detection of F-actin

Following a 24 h co-treatment with 15 µg/mL PAN and engineered exosomes, the cells were subjected to cytoskeleton staining. To visualize the cytoskeleton, MPC-5 cells were fixed at room temperature for 15 min and then stained with Actin-Tracker Green, which specifically labels microfilaments. DAPI staining was then carried out for 10 min. Fluorescence and DAPI signals were subsequently detected using a Leica STELLARIS STED confocal microscope.

Biodistribution of RAPA@MEX-PL In Vivo

To evaluate the biodistribution of the different formulations, RAPA, RAPA@MEX, and RAPA@MEX-PL were labeled with Cy5 and excess dye or antibody was removed by ultrafiltration (3 cycles). The dose of RAPA was 1 mg/kg in all formulations. For the nanoparticle groups, the MEX carrier dose was 10.50 mg/kg. The labeled drug was injected intravenously into MRL/lpr and MRL/MPJ mice. An AniView Pro animal imaging system (BLT Biotechnology Co., Ltd.) enabled tracking of drug biodistribution via NIRF (near-infrared fluorescence) at designated time points across a 24-hour window. At 4 h and 24 h post-administration, the animals were euthanized, and their principal organs were collected for ex vivo NIRF imaging. The imaging system's native software provided a tool for fluorescence quantification. Using this tool, we determined the average intensity per sample. In addition, OCT embedding and frozen sectioning of fresh heart, lung, liver, kidney, lymph nodes, and spleen were performed using an HM525 NX automatic cryostat (Epredia). Meanwhile,

renal tissue sections were stained overnight with COL4A3 primary antibody, incubated with secondary antibody, and nucleated with DAPI. Tissue sections were visualized with a Leica STELLARIS STED confocal microscope following 10 min of DAPI staining.

MRL/lpr Spontaneous SLE Mouse Model

MRL/lpr mice (16 weeks old) were randomized into 8 groups: 7 treatment groups receiving weekly IV injections of PBS, MSC, MEX, RAPA, MEX-PL, RAPA@MEX (RM), or RAPA@MEX-PL (RMP) for 4 weeks, plus one healthy control (MRL/MPJ). The dose of RAPA was 1 mg/kg in all formulations. For the nanoparticle groups, the MEX carrier dose was 10.50 mg/kg. Body weight was monitored weekly. Post-treatment, urinary protein was quantified via BCA assay. Spleen and axillary lymph nodes were weighed. Kidneys underwent H&E and immunohistochemical staining. Blood was analyzed for hepatic/renal function, serum creatinine, anti-dsDNA antibodies, and cytokines (ELISA). Splenocytes were analyzed by flow cytometry using F4/80-PE, CD86-PE/Cy7, CD11B-FITC, CD206-APC, and LY-6G-PerCP/Cy5.5. The Animal Ethics Committee of the First Affiliated Hospital of Anhui Medical University granted approval for all animal procedures (Approval No. IACUC-2502029).

Extraction and Measurement of cfDNA

CfDNA was isolated from mouse serum (MRL/MPJ and MRL/lpr mice) with a commercial nucleic acid extraction kit. The concentration of the isolated cfDNA was subsequently determined using a Nanodrop 2000 spectrophotometer (Thermo Fisher).

Evaluation of the Biocompatibility of RAPA@MEX-PL

After the treatment, blood samples were collected to measure the levels of liver functions. Potential pathological damage to the major organs was assessed by H&E staining.

Evaluation of Renal Histopathology

The renal tissue was examined under a light microscope to assess glomerulonephritis through H&E staining. To visualize and quantify immune complex deposition, tissue sections were stained overnight with primary antibodies (mouse IgG, C3, nephrin or

WT-1), followed by secondary antibody incubation and DAPI nuclear counterstaining. Quantitative analysis of glomerular fluorescence intensity for IgG (red) and C3 (green), nephrin (red) and WT-1 (green) was performed using ImageJ software.

Statistical Analysis

Biological replicates ($n \geq 3$) were incorporated into all experimental conditions. Data are mean ± SD, analyzed with GraphPad Prism 9.0. For comparisons between groups, one-way ANOVA with Tukey's correction was applied. Significance levels: *$p < 0.05$, **$p < 0.01$, ***$p < 0.001$.

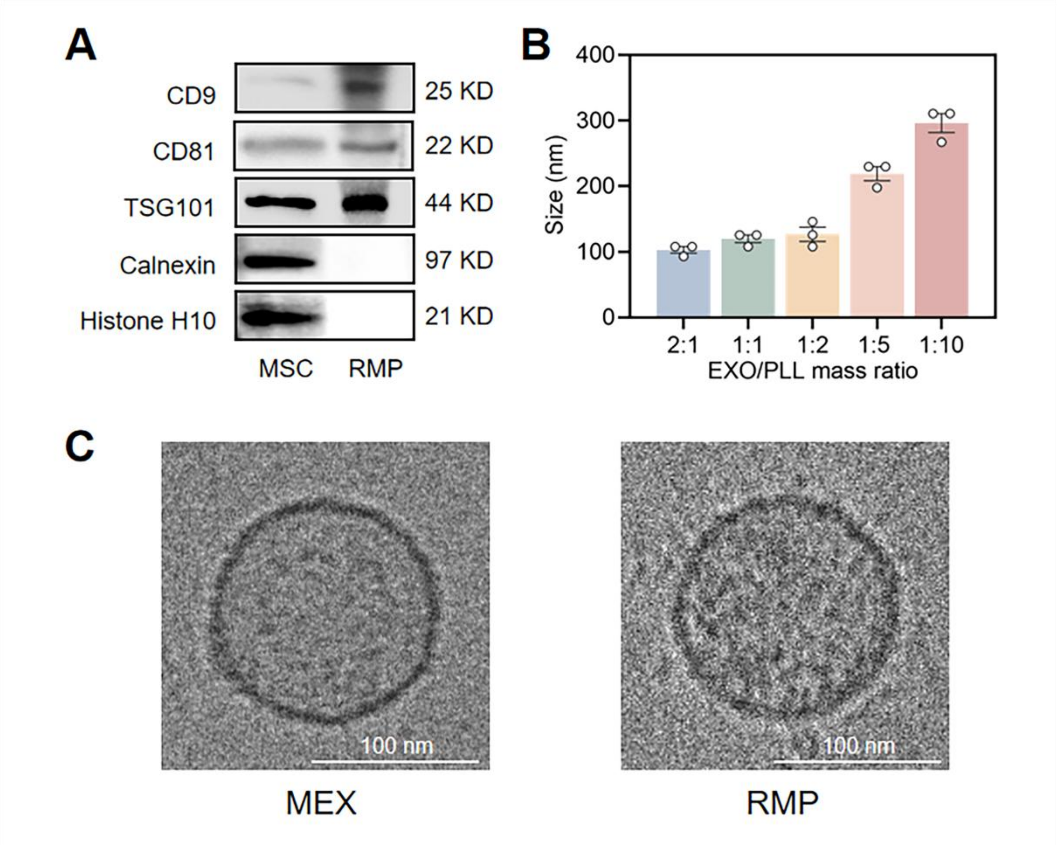

Figure S1. Characterization of engineered exosomes. (A) Detection of exosomal markers (CD9, CD81, TSG101) and negative markers (Calnexin, Histone H10) by Western blotting. (B) Particle size distribution of exosomes and PLL at varying mass ratios. Data are mean ± SD (n = 3). (C) Cryo-transmission electron microscopy (cryo-TEM) morphological analysis of exosomes (MEX) and engineered exosomes (RMP). Scale bar, 100 nm.

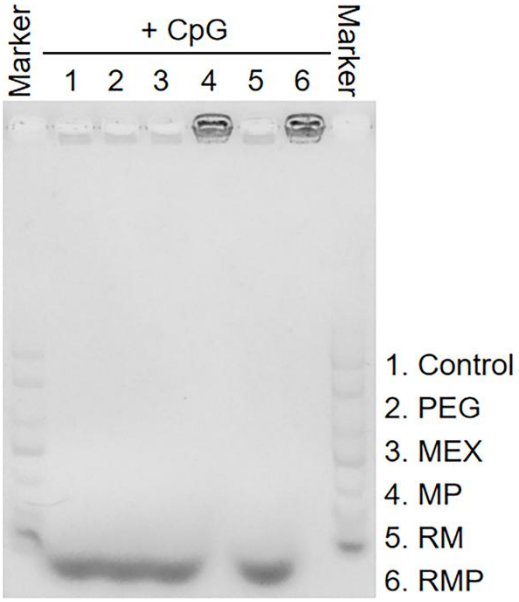

Figure S2. DNA binding potency of engineered exosomes. Agarose gel electrophoresis image of CpG after 24 h incubation separately with each group.

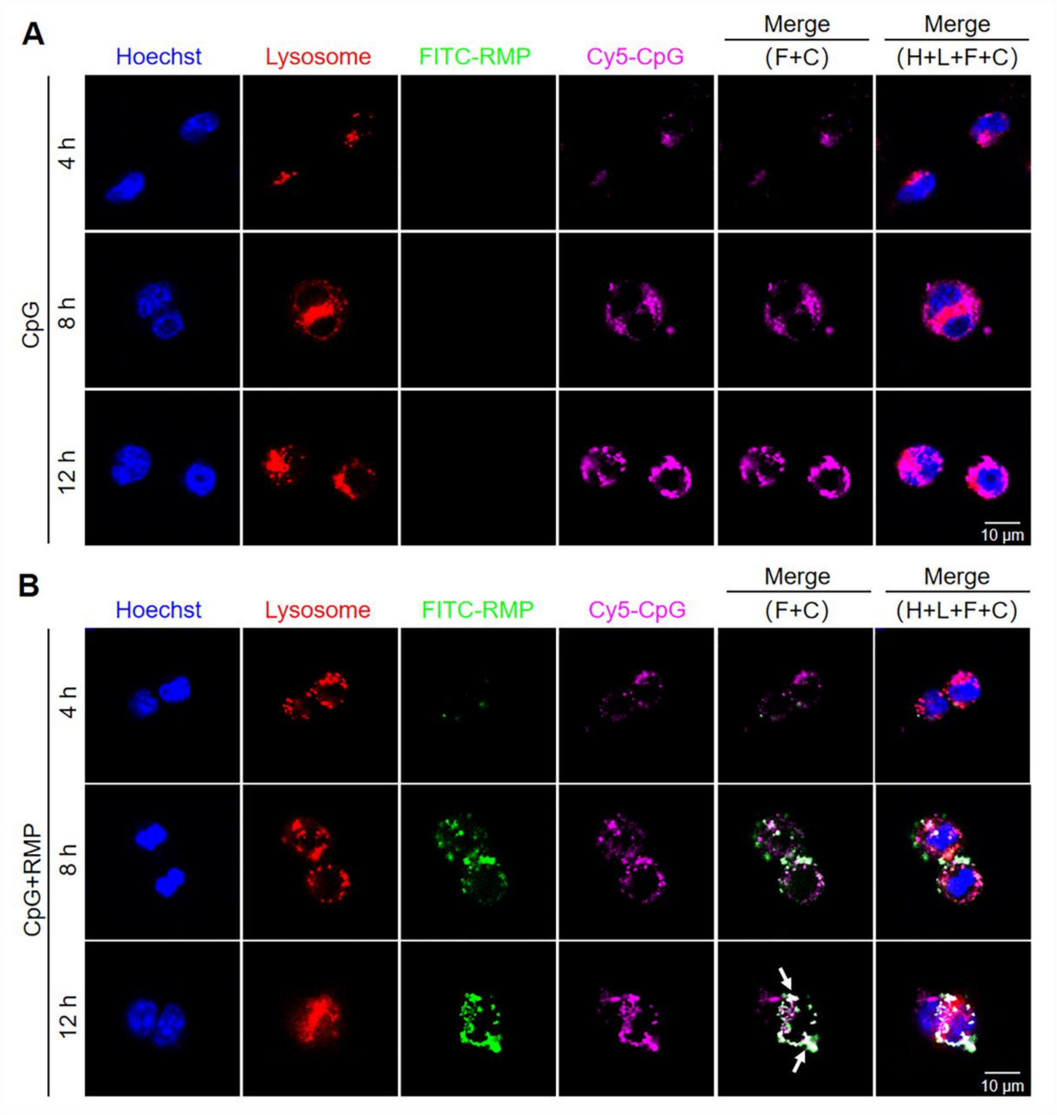

Figure S3. Evaluation of trafficking of engineered exosomes and the immuno-stimulatory nucleic acids at different time points. RAW264.7 macrophages were co-treated with CpG and RMP simultaneously. (A) Evaluation of the immuno-stimulatory nucleic acids at various time intervals. Scale bar, 10 μm. (B) Evaluation of colocalization of engineered exosomes RMP with the nucleic acids at multiple time points. Scale bar, 10 μm.

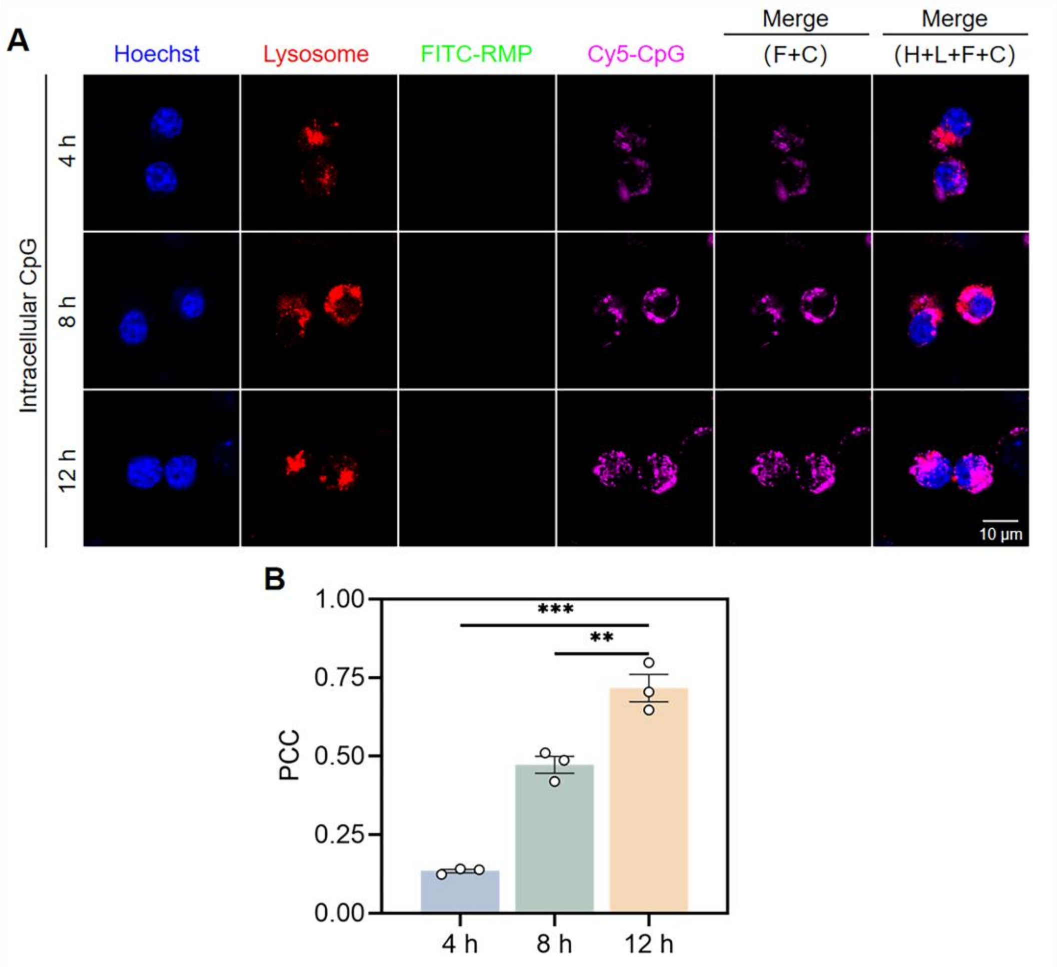

Figure S4. Time-dependent analysis of intracellular trafficking of immunostimulatory nucleic acids. (A) Time-course analysis of CpG internalization in cells. Evaluation of the internalized immunostimulatory nucleic acids at multiple time points. Scale bar, 10 μm. (B) A progressive increase over time in the colocalization of FITC-RMP and Quasar 670-CpG was observed in RAW264.7 macrophages by Pearson's correlation coefficient (PCC) analysis, following a 4-hour pre-exposure to CpG (mean ± SD, n = 3). A p-value of < 0.05 was considered statistically significant (***$p < 0.001$).

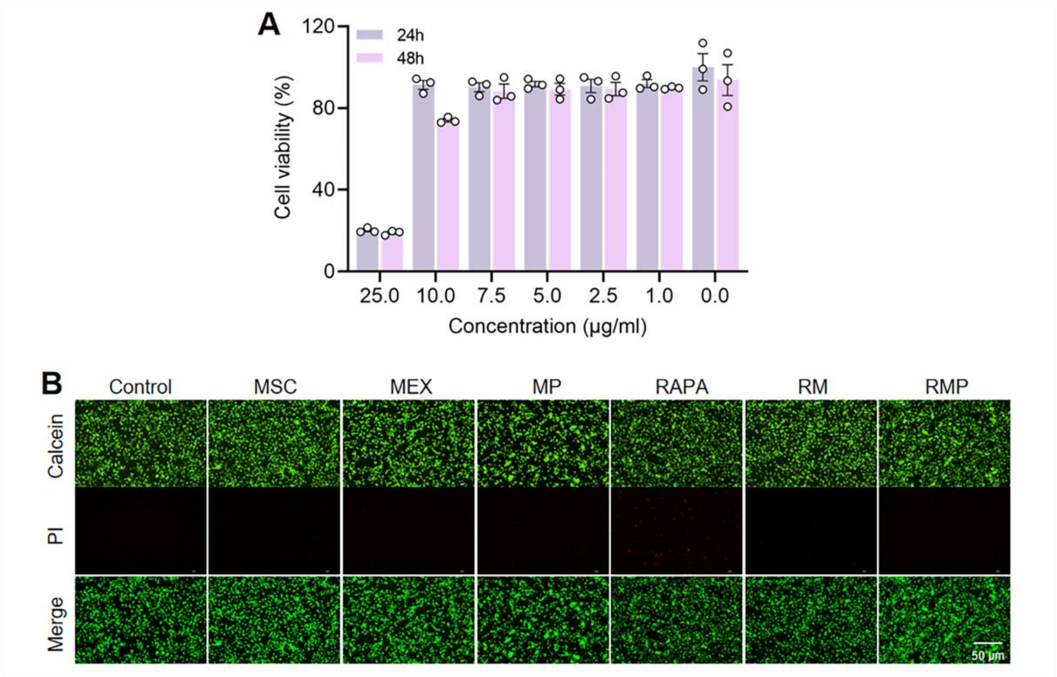

Figure S5. Engineered exosomes displayed negligible cytotoxicity in macrophages. (A) In vitro experiments, the cytotoxicity curve of engineered exosomes (with concentration referenced to RAPA concentration) was tested in macrophages (mean ± SD, n = 3). (B) Live and dead cell staining was performed on cells following a 24 h treatment period with different agents. Scale bar, 50 μm.

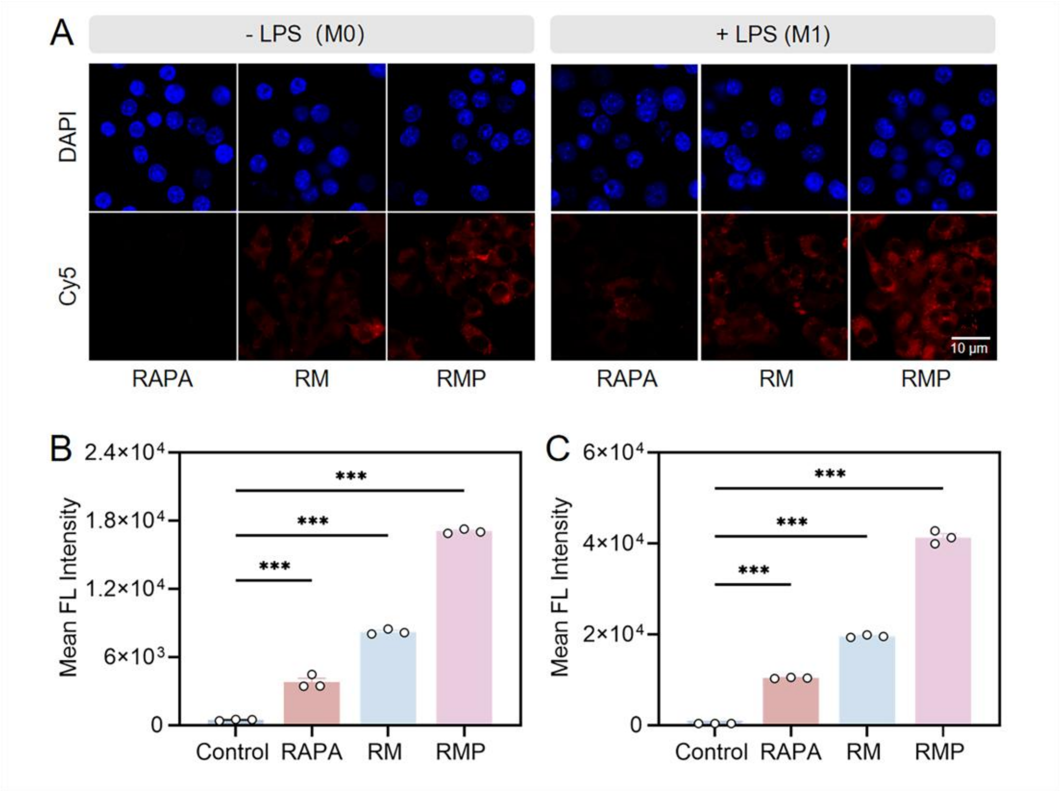

Figure S6. Efficient uptake of engineered exosomes by macrophages. (A) Confocal fluorescence microscopy images of cellular uptake of engineered exosomes in RAW264.7 cell lines for 60 min. Scale bar, 10 μm. (B) and (C) Statistical plot of macrophages uptake from flow cytometry (mean ± SD, n = 3). A *p*-value of < 0.05 was considered statistically significant (***$p < 0.001$).

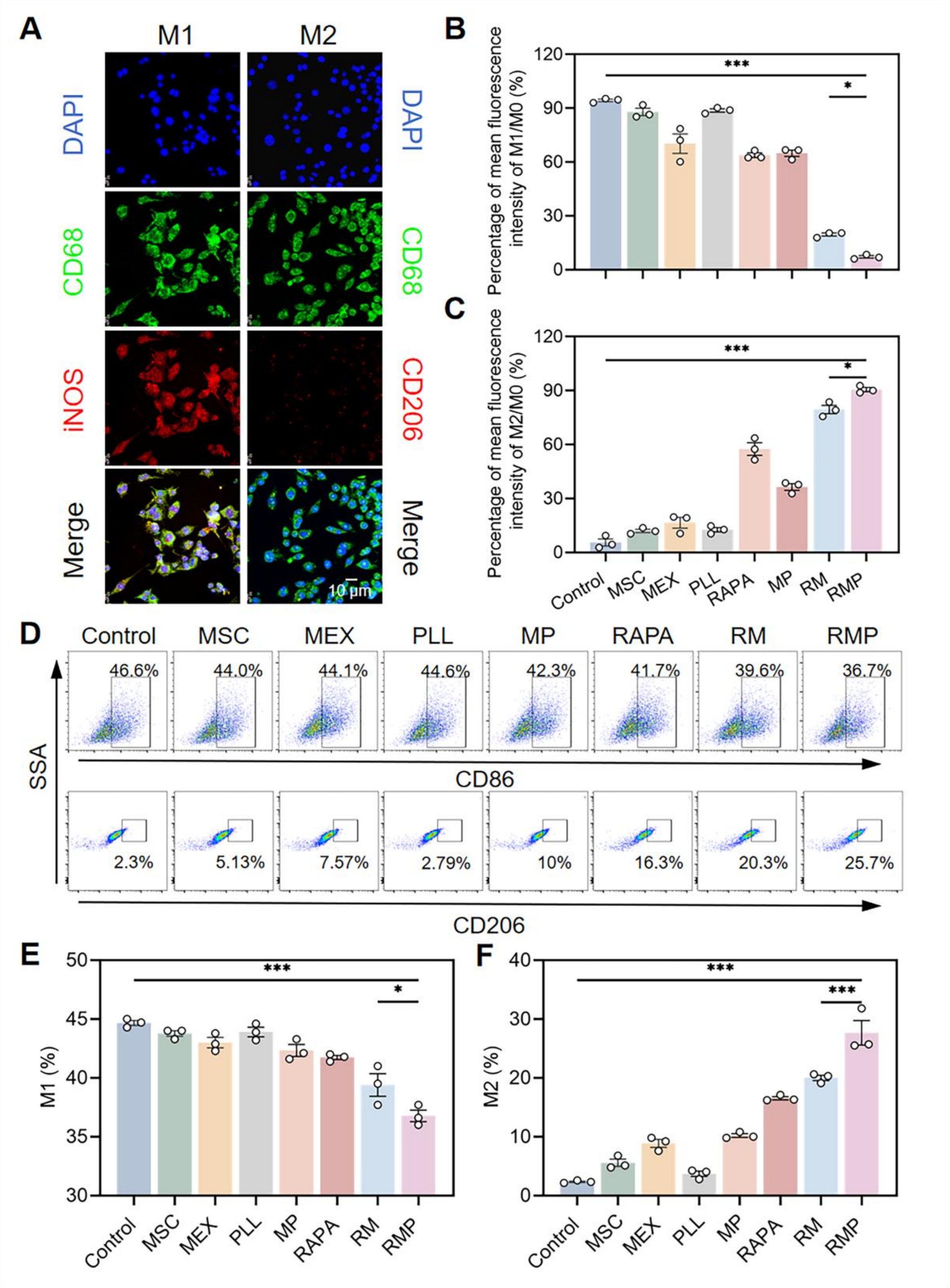

Figure S7. Engineered exosomes modulated the overactivated immune imbalance. (A) Representative immunofluorescence images of macrophage polarization after 24 h of PLL treatment. Macrophages were stained for CD68 (green), iNOS (red), and CD206 (red). DAPI counterstaining (blue) labeled cell nuclei. Scale bar, 10 µm. (B) and (C) Quantitative ratio analysis of M1 and M2 immunofluorescence intensity (mean ± SD, n = 3). (D) Flow cytometric profiling of macrophage polarization states. (E) and (F) Statistical plots of M1 and M2 proportions in each group (mean ± SD, n = 3). A *p*-value of < 0.05 was considered statistically significant (****p* < 0.001).

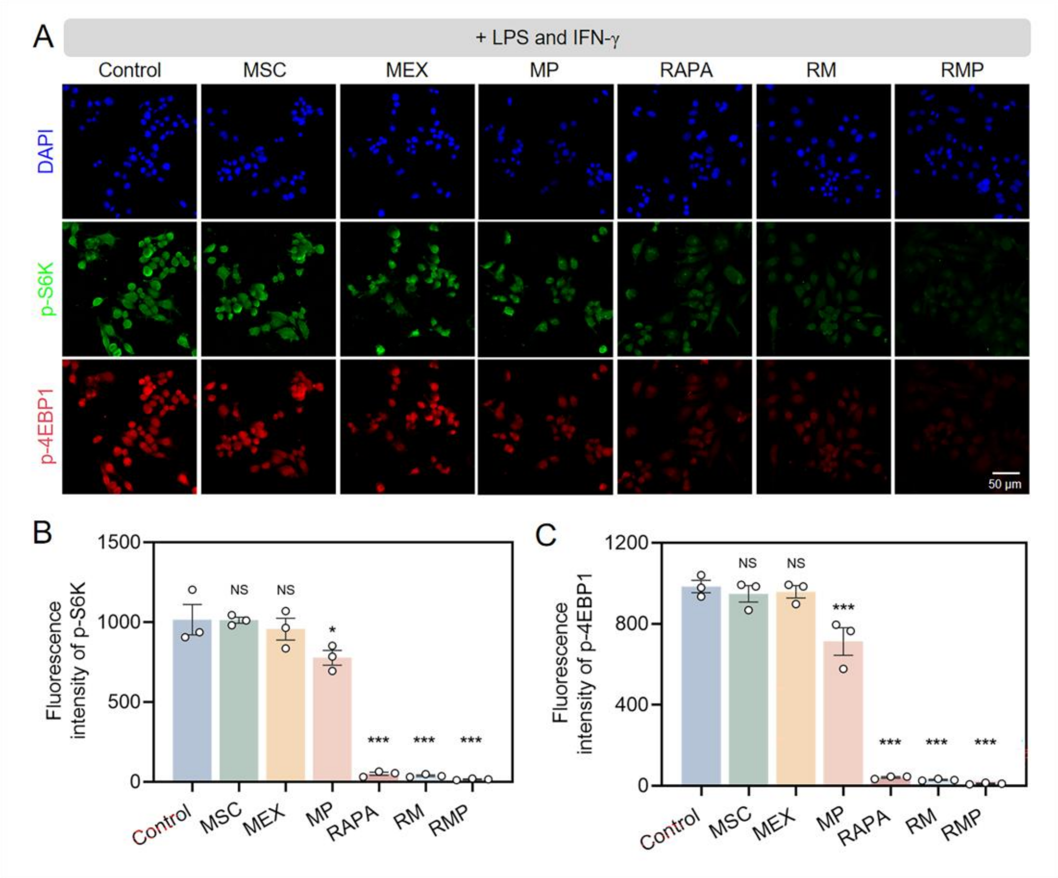

Figure S8. Immunofluorescence analysis of mTOR inhibition. (A) Representative immunofluorescence images of macrophages stained for p-4EBP1 (red), p-S6K (green). DAPI (blue) served as nuclear counterstain. Scale bar, 50 μm. (B) and (C) Quantitative assessment of the fluorescence intensities corresponding to p-S6K and p-EBP1 (mean ± SD, n = 3). A *p*-value of < 0.05 was considered statistically significant (****p* < 0.001).

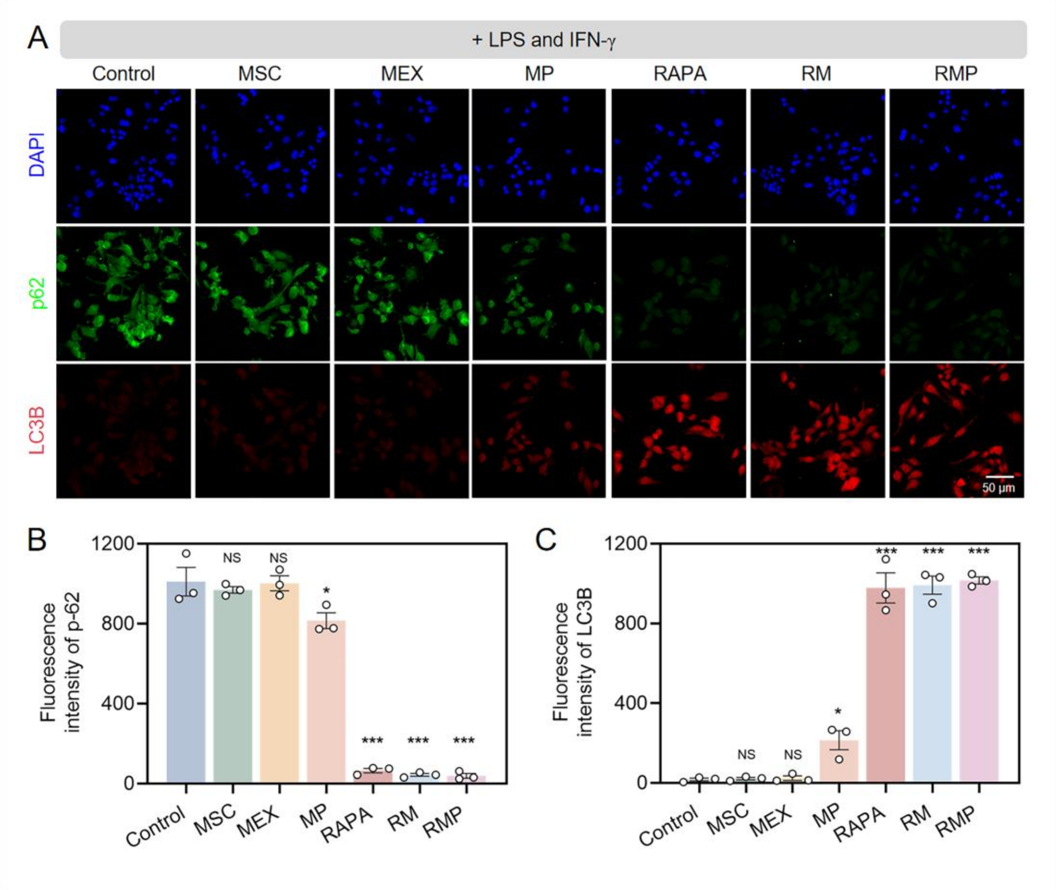

Figure S9. Immunofluorescence analysis of autophagy flux. (A) Representative immunofluorescence images of macrophages stained for p62 (green), LC3B (red), and DAPI counterstaining (blue) labeled cell nuclei. Scale bar, 50 μm. (B) and (C) Quantitative assessment of the fluorescence intensities corresponding to p62 and LC3B (mean ± SD, n = 3). A *p*-value of < 0.05 was considered statistically significant (***$p < 0.001$).

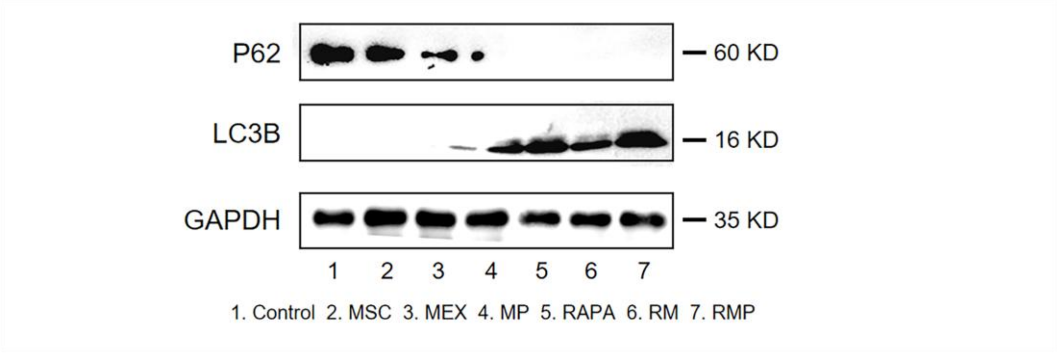

Figure S10. Western blot analysis of autophagy flux. Representative images depicting P62 and LC3B protein expression in macrophages under the indicated treatment conditions.

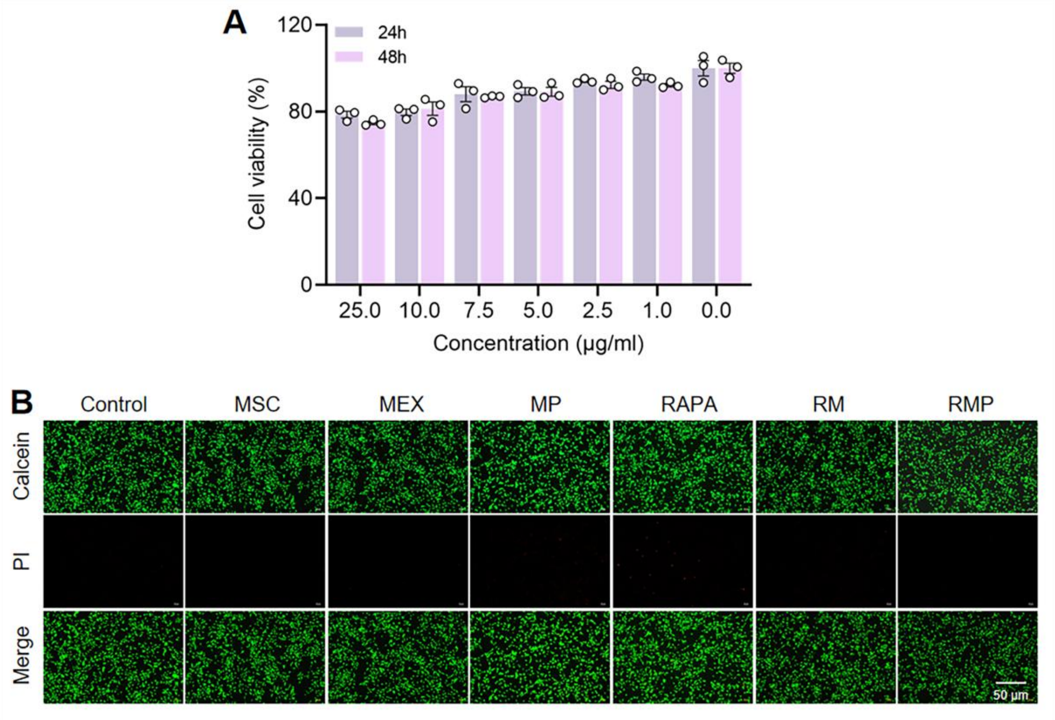

Figure S11. Engineered exosomes were virtually non-toxic to podocytes. (A) In vitro experiments, the cytotoxicity curve of engineered exosomes (with concentration referenced to RAPA concentration) was tested in MPC-5 (mean ± SD, n = 3). (B) Treated with different treatments for 24 h were stained with live and dead cells. Scale bar, 50 μm.

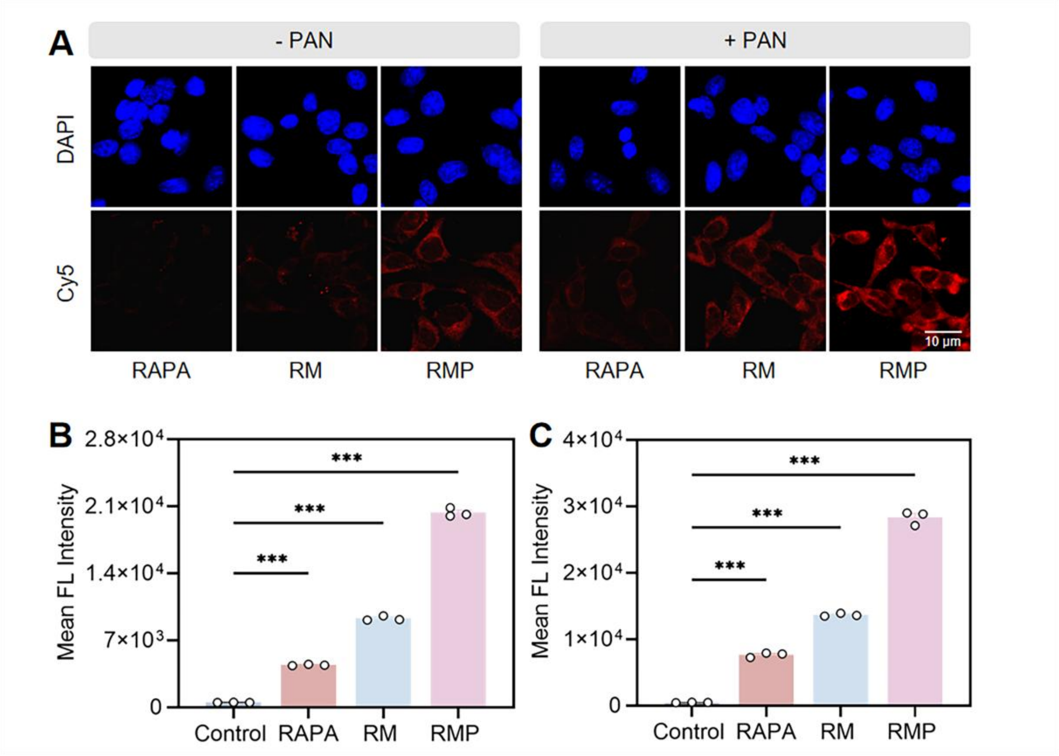

Figure S12. Efficient uptake of engineered exosomes by podocytes. (A) Confocal fluorescence microscopy images and of cellular uptake of engineered exosomes in podocytes (MPC-5) for 60 min. Scale bar, 10 μm. (B) and (C) Statistical plot of podocyte uptake from flow cytometry (mean ± SD, n = 3). A *p*-value of < 0.05 was considered statistically significant (***$p < 0.001$).

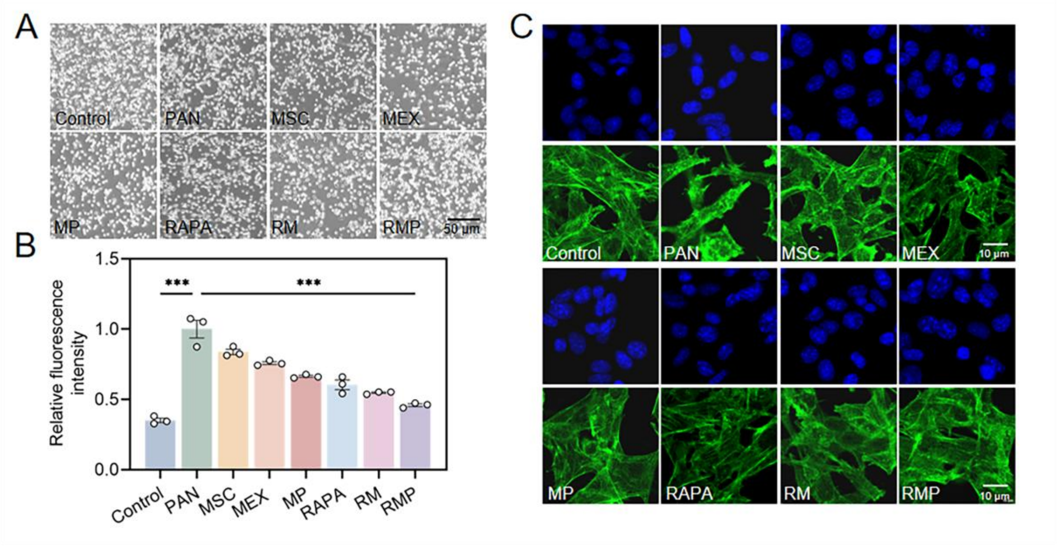

Figure S13. Engineered exosomes effectively protected podocyte function. (A) DCFH-DA staining for ROS generation of inflammatory MPC-5 using fluorescence microscopy. Scale bar, 50 μm. (B) Graphical representation of ROS fluorescence quantification (mean ± SD, n = 3). (C) Morphology and light microscopy images of MPC-5 cells. Scale bar, 10 μm. A *p*-value of < 0.05 was considered statistically significant (***$p < 0.001$).

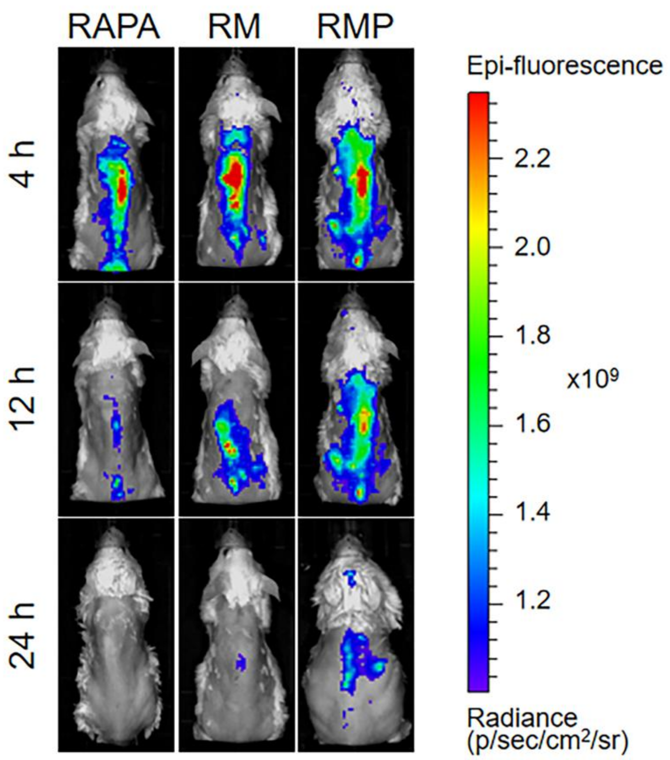

Figure S14. In vivo biodistribution of engineered exosomes in MRL/MPJ mice. Biodistribution of Cy5-labeled RAPA, RM and RMP in MRL/MPJ mice at different time points post-injection.

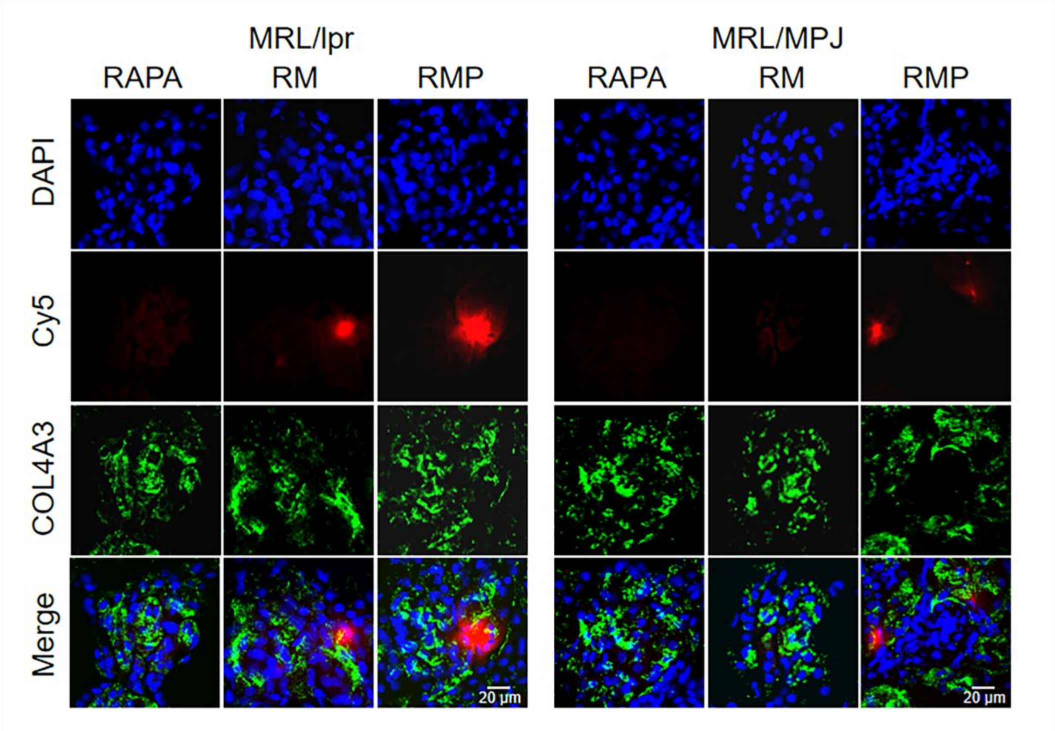

Figure S15. The inflammatory microenvironment in diseased kidneys promotes the selective accumulation of engineered exosomes. Colocalization analysis of Cy5-labeled formulations with the glomerular basement membrane. Representative immunofluorescence images of kidney sections from mice administered with Cy5-RAPA, RM and RMP. Glomerular basement membrane was stained with collagen IV (green). Scale bar, 20 μm.

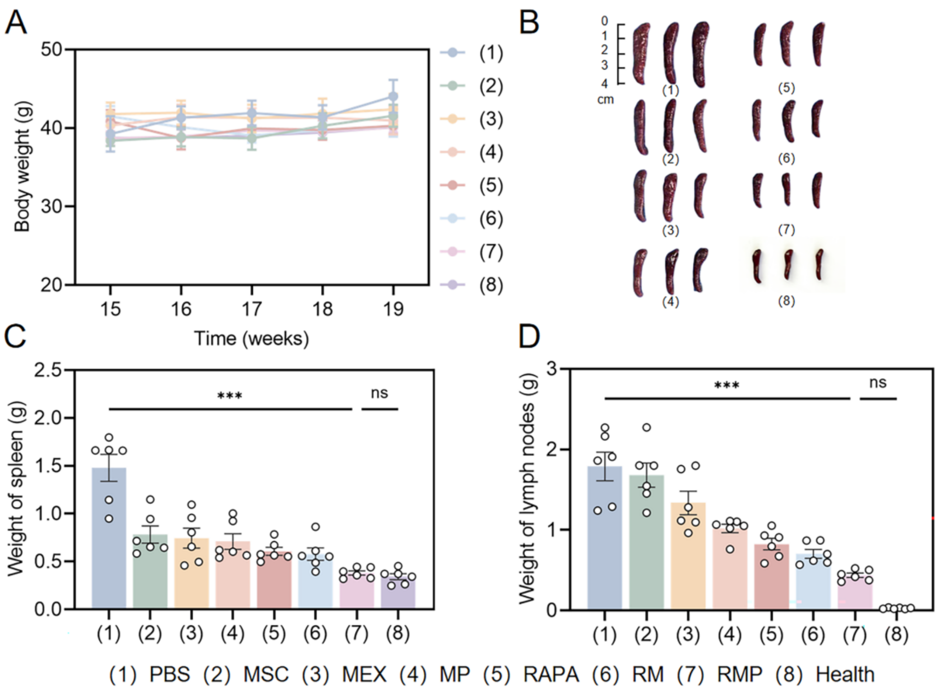

Figure S16. Engineered exosomes effectively improved the condition of lupus mice. (A) Body weight variations in mice during the pre-treatment and post-treatment periods (mean ± SD, n = 6). (B) Representative splenic photographs from mice in all groups taken at week 19. (C) and (D) Weights of spleens and lymph nodes harvested from mice in each group at week 19 (mean ± SD, n = 6). A *p*-value of < 0.05 was considered statistically significant (****p* < 0.001).

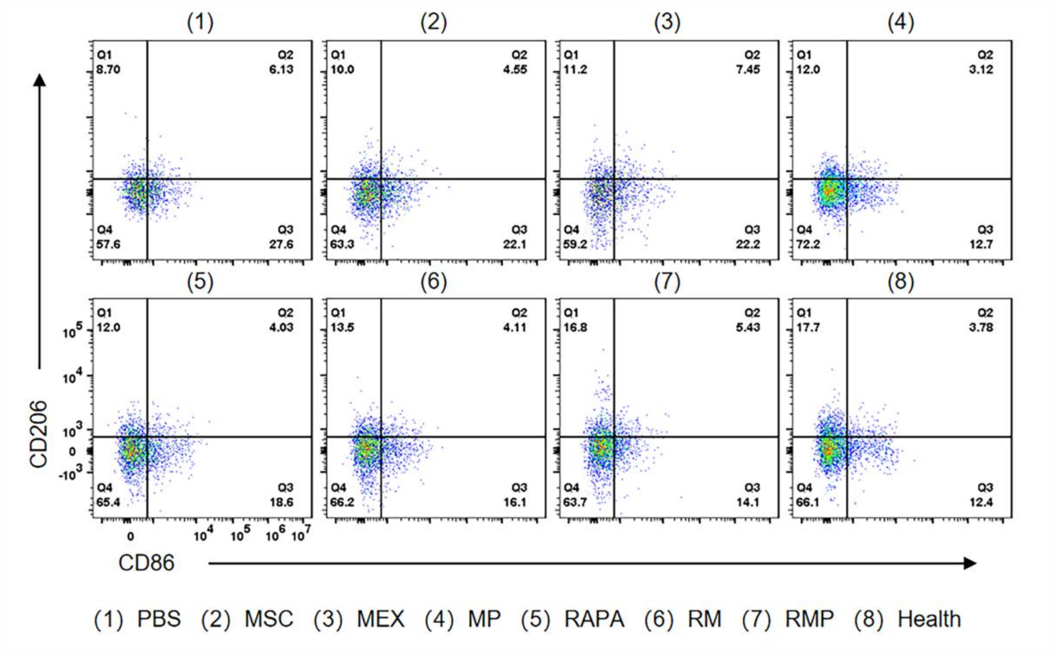

Figure S17. Engineered exosomes significantly alleviated renal lesions in lupus mice. Flow cytometric evaluation of splenic macrophage polarization in mice at the 19-week time point.

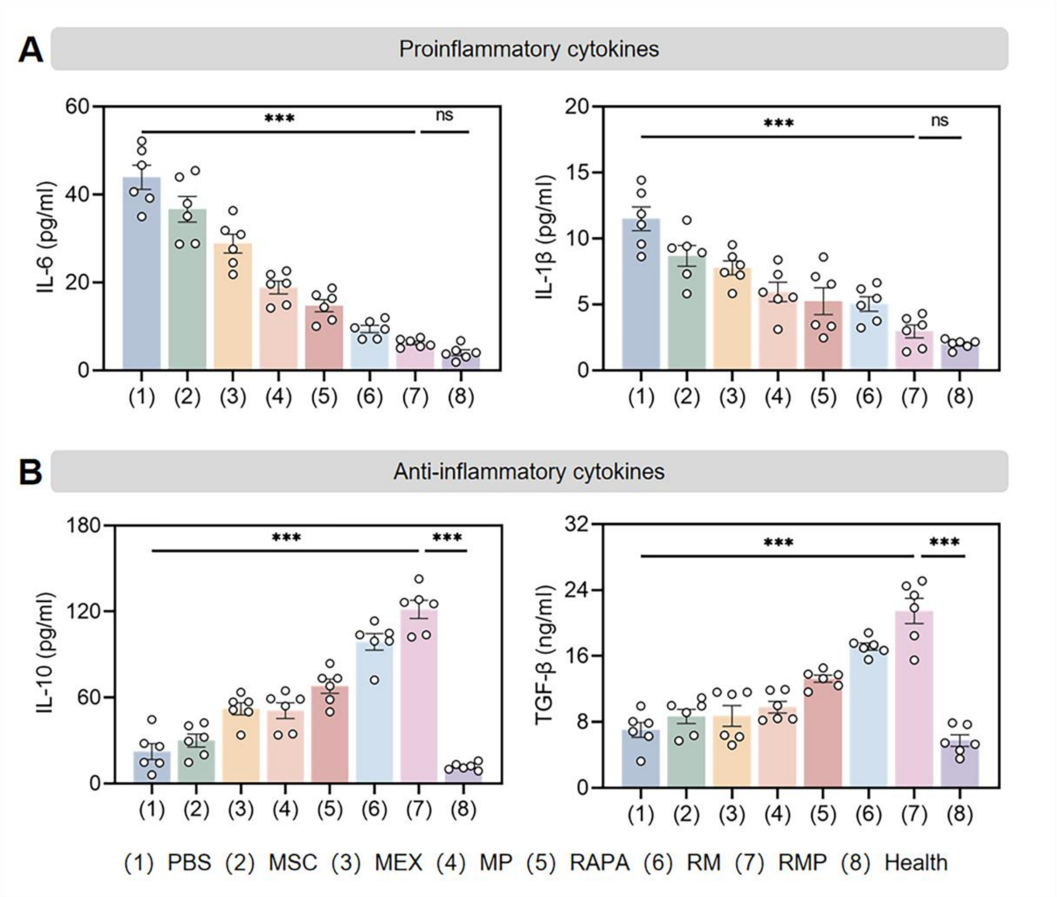

Figure S18. Engineered exosomes effectively improved the condition of lupus mice. (A) and (B) Quantification of pro-inflammatory and anti-inflammatory cytokines secreted in mouse serum at week 19 was performed using enzyme-inked immunosorbent assay (mean ± SD, n = 6). A *p*-value of < 0.05 was considered statistically significant (****p* < 0.001).

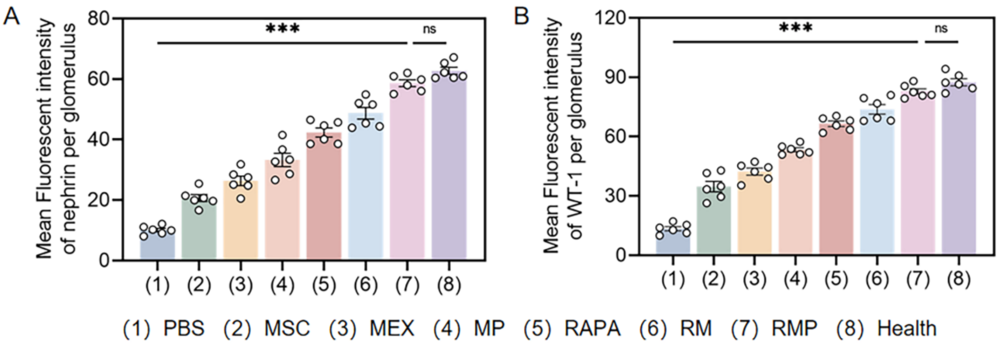

Figure S19. Engineered exosomes effectively protect podocytes in lupus mice. (A) and (B**)** Quantitative assessment of the mean fluorescent intensity of nephrin and WT-1 per glomerulus (mean ± SD, n = 6). A *p*-value of < 0.05 was considered statistically significant (****p* < 0.001).

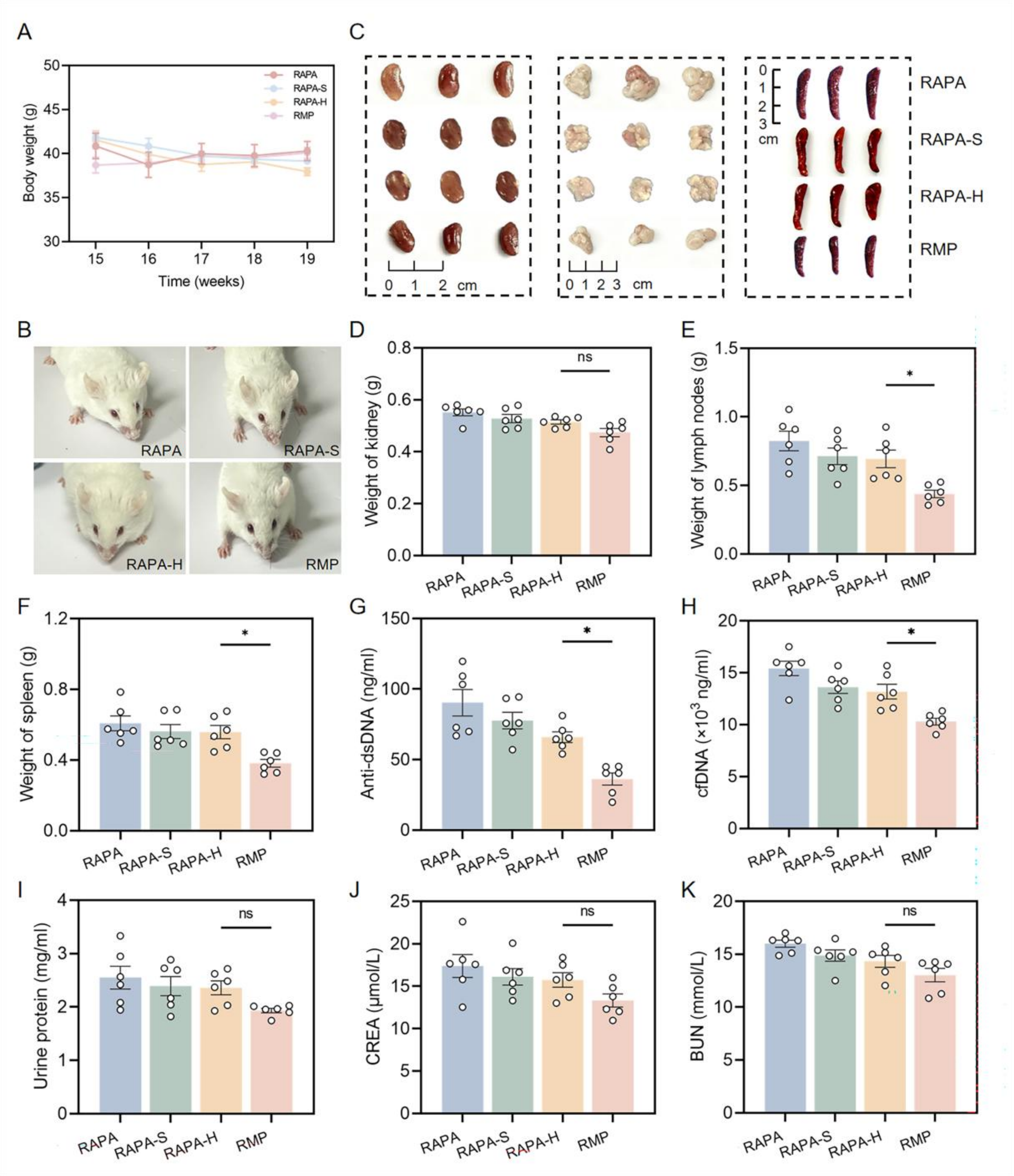

Figure S20. Cationic exosomes enhance the efficacy and reduce the toxicity of rapamycin in lupus-prone mice. (A) Body weight changes at 5 weeks in lupus mice from different treatment groups. (B) Representative facial photographs of mice after the treatment period. (C-F) Representative pictures and weight of kidney, lymph nodes and spleen after different treatments (mean ± SD, n = 6). (G) Serum dsDNA levels of the four groups of mice at week 19 (mean ± SD, n = 6). (H) Serum cfDNA levels in four groups of lupus mice at week 19 (mean ± SD, n = 6). (I-K) The urine protein, creatinine and BUN concentration change in the four groups at 19 weeks (mean ± SD, n = 6) (RAPA-Standard, RAPA-S; RAPA-High, RAPA-H). A *p*-value of < 0.05 was considered statistically significant (****p* < 0.001).

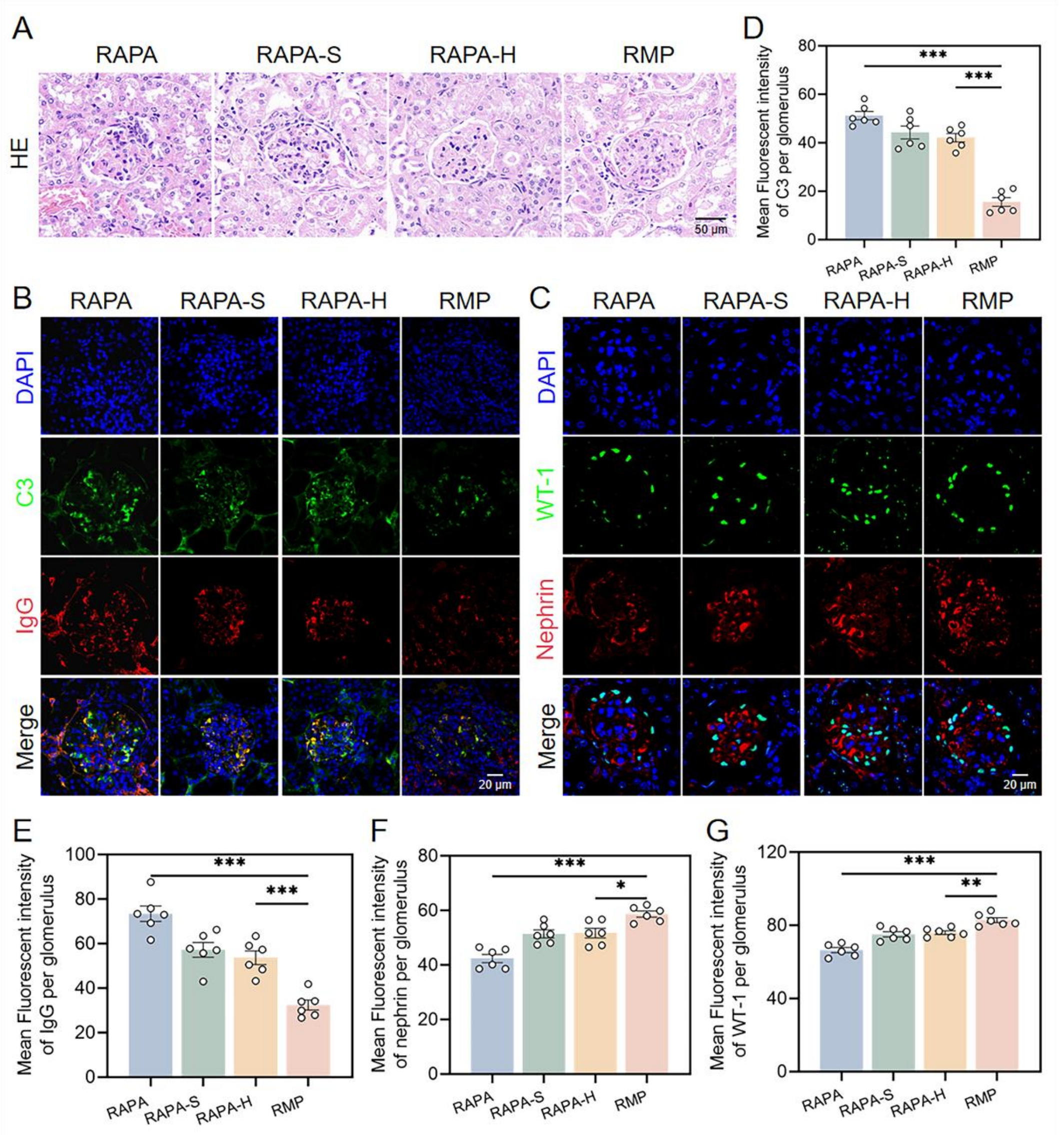

Figure S21. Cationic exosomes delivering rapamycin show superior therapeutic effects in lupus nephritis compared to rapamycin alone. (A) Representative micrographs of H&E-stained renal sections harvested from four mouse groups. Scale bar, 20 μm. (B) Immunofluorescence staining revealing C3 (green) and IgG (red) in the kidneys of lupus mice. Scale bar, 20 μm. (C) Representative immunofluorescence micrographs of nephrin (red) and WT-1 (green) in renal tissues of lupus mice. Scale bar, 20 μm. (D) and (E) Quantitative assessment of the mean fluorescence intensity of C3 and IgG in the positive areas of individual glomeruli (mean ± SD, n = 6). (F) and (G) Quantitative of the mean fluorescence intensity for nephrin and WT-1 within individual glomerular positive areas (RAPA-S, RAPA-Standard; RAPA-H, RAPA-High) (mean ± SD, n = 6). Statistical significance was defined as $p < 0.05$ (*$p < 0.05$ and ***$p < 0.001$).

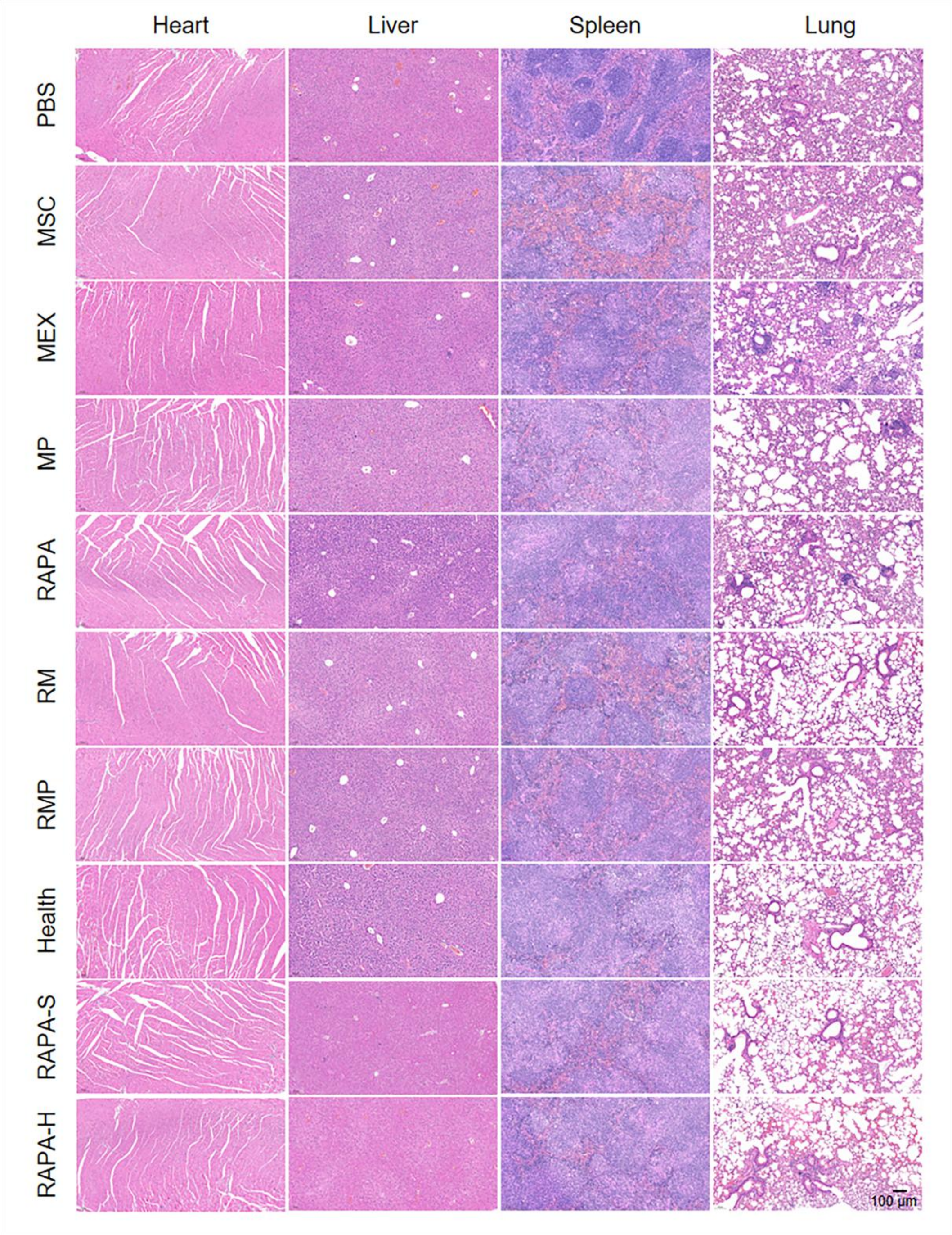

Figure S22. H&E staining was performed on major organs harvested from mice across different treatment groups. Representative H&E-stained pathological sections of heart, liver, spleen, and lung tissues from ten mouse groups. Scale bar, 100 μm. (RAPA-S, RAPA-Standard; RAPA-H, RAPA-High).

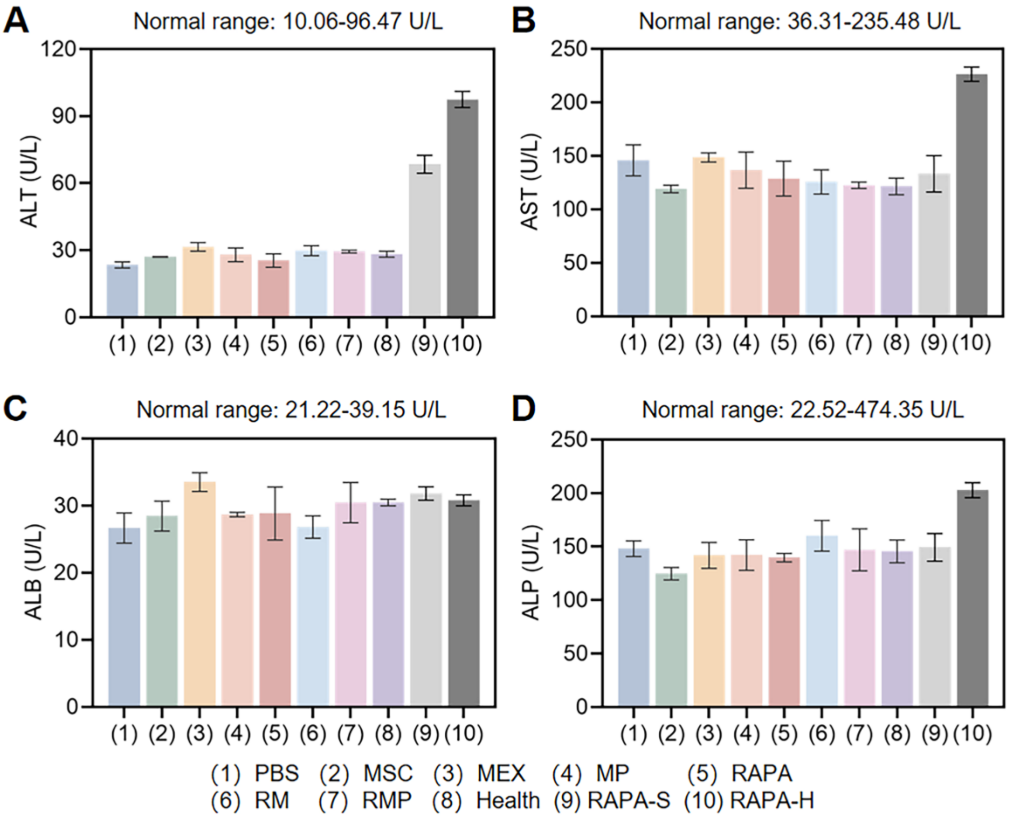

Figure S23. Assessment of liver function in mice after different treatments. (A-D) Bar charts of liver function related indicators of the ten groups of mice (RAPA-S, RAPA-Standard; RAPA-H, RAPA-High) (mean ± SD, n = 3).
